# Borderline personality disorder: associations with psychiatric disorders, somatic illnesses, trauma, and adverse behaviors

**DOI:** 10.1038/s41380-022-01503-z

**Published:** 2022-03-18

**Authors:** Ashley E. Tate, Hanna Sahlin, Shengxin Liu, Yi Lu, Sebastian Lundström, Henrik Larsson, Paul Lichtenstein, Ralf Kuja-Halkola

**Affiliations:** 1 Department of Medical Epidemiology and Biostatistics, Karolinska Institutet, Solna, Sweden; 2 Centre for Psychiatry Research, Department of Clinical Neuroscience, Karolinska Institutet, Stockholm, Sweden; 3 Centre for Ethics, Law and Mental Health (CELAM), University of Gothenburg, Gothenburg, Sweden; 4 Gillberg Neuropsychiatry Centre, University of Gothenburg, Gothenburg, Sweden; 5 School of Medical Sciences, Örebro University, Örebro, Sweden

**Keywords:** Psychiatric disorders, Depression, Addiction, Bipolar disorder, Schizophrenia

## Abstract

In one of the largest, most comprehensive studies on borderline personality disorder (BPD) to date, this article places into context associations between this diagnosis and (1) 16 different psychiatric disorders, (2) eight somatic illnesses, and (3) six trauma and adverse behaviors, e.g., violent crime victimization and self-harm. Second, it examines the sex differences in individuals with BPD and their siblings. A total of 1,969,839 Swedish individuals were identified from national registers. Cumulative incidence with 95% confidence intervals (CI) was evaluated after 5 years of follow-up from BPD diagnosis and compared with a matched cohort. Associations were estimated as hazard ratios (HR) with 95% CIs from Cox regression. 12,175 individuals were diagnosed with BPD (85.3% female). Individuals diagnosed with BPD had higher cumulative incidences and HRs for nearly all analyzed indicators, especially psychiatric disorders. Anxiety disorders were most common (cumulative incidence 95% CI 33.13% [31.48–34.73]). Other notable findings from Cox regressions include psychotic disorders (HR 95% CI 24.48 [23.14–25.90]), epilepsy (3.38 [3.08–3.70]), violent crime victimization (7.65 [7.25–8.06]), and self-harm (17.72 [17.27–18.19]). HRs in males and females with BPD had overlapping CIs for nearly all indicators. This indicates that a BPD diagnosis is a marker of vulnerability for negative events and poor physical and mental health similarly for both males and females. Having a sibling with BPD was associated with an increased risk for psychiatric disorders, trauma, and adverse behaviors but not somatic disorders. Clinical implications include the need for increased support for patients with BPD navigating the health care system.

## Introduction

Borderline Personality Disorder (BPD; Diagnostic and Statistical Manual of Mental Disorders [DSM] terminology), or Emotionally Unstable Personality Disorder (International Classification of Diseases [ICD] terminology), is a serious psychiatric disorder estimated to affect 1.7% of the worldwide population [1]. The core features of this diagnosis include: unstable interpersonal relationships, recurring self-harm, and emotional dysregulation [2].

Receiving a BPD diagnosis has been repeatedly associated with a high degree of psychiatric and somatic comorbidities, traumatic events, and criminal behavior [3–5]. These results have shaped the clinical perception and research directions of BPD, however specific estimates across psychiatric disorders, somatic illnesses, trauma, and adverse behavior have never been comprehensively presented in one study. Moreover, the majority of past studies were limited to smaller clinical samples or cross-sectional community samples, which did not allow for unselected samples nor longitudinal data. Additional longitudinal trait-based epidemiological studies have examined BPD symptoms in twins, however, few participants reached the full diagnostic criteria and thus may not be reflective of individuals with severe BPD symptoms [6, 7]. Therefore, it is of interest to examine these estimates in a representative and well-powered population-based study in order to provide the context for a BPD diagnosis and reframe misconceptions.

### Psychiatric disorders

Psychiatric comorbidities are the rule rather than the exception in patients with BPD. A Swedish national study reported that 95.7% of individuals with a BPD diagnosis had a comorbid psychiatric diagnosis [8]. Mood disorders, post-traumatic stress disorder (PTSD), impulsive disorders, and bipolar disorders are commonly associated with BPD symptoms and diagnosis [9–11]. Precise estimates for these comorbidities are lacking, especially for rare and serious disorders, e.g., psychotic disorders [12].

### Somatic illnesses

Individuals with BPD use health care services at a higher rate than those with other personality disorders, and even elevated BPD symptoms are associated with receiving disability [13, 14]. BPD has been linked to poor somatic health, such as obesity, diabetes, gastrointestinal disease, cardiovascular disease, hypertension, chronic pain, and sexually transmitted infections [3, 15]. It is likely that somatic illnesses associated with BPD have been overlooked in smaller studies, for example, infertility [16, 17]. Moreover, patients often perceive the severity of their illness worse than reports based on medical records, highlighting the importance of objective measurement [18].

### Trauma and adverse behaviors

Up to 90% of patients with BPD are estimated to have a history of childhood trauma [1]. BPD has been linked to an increased risk for sexual abuse victimization in adulthood [19] and physical trauma resulting from accidents [3]. However, the association between BPD and other trauma types, e.g., death of a family member, is unclear. The relationship between trauma and BPD is theorized to be bidirectional, although the evidence for this is conflicting [20]. Similar to somatic reports, capturing objective measures of trauma would prevent biases that may arise from self-reports [21].

The association between violent crime and a BPD diagnosis is well documented, however less literature exists on nonviolent offenses [22]. It is unclear which nonviolent offenses are predominant in patients with BPD, although it is likely borne from impulsive actions. As recurrent self-harm is a core feature of BPD [2], we expect the rate of self-harm requiring medical attention to be higher than the general population. However, a precise estimate from a population sample is unknown.

### Sex differences

BPD is predominately diagnosed in females, although evidence suggests that this is largely the result of a diagnostic bias [23]. Moreover, gender differences have been found for symptom expression and comorbidities [22]. Males have a higher prevalence of substance use disorders and antisocial personality disorder and exhibit symptoms related to aggression; while females show increased rates of risky behavior and an increased prevalence of comorbid mood disorders, eating disorders, and PTSD [22, 24]. Males are typically underrepresented in BPD studies, thus potential differences in outcomes and precursors of BPD is of particular importance. Given a diagnostic bias, males would need greater symptom severity in order to be diagnosed, leading to a difference in symptom severity between the sexes. With this, we hypothesize elevated rates across most categories for males with BPD compared to their female counterparts.

Further, this raises etiological questions about the differences between the sexes. With our hypothesis that males with BPD will have more severe symptomatology, we postulate that individuals with a brother diagnosed with BPD will have higher rates of diagnoses and adverse outcomes across all domains compared to those with a sister with a BPD diagnosis, similar to the so-called female protective effect in autism, with the sexes reversed [25].

### Present study

The primary aim of this study was to describe the extent of the association for individuals with BPD and (1) psychiatric disorders, (2) somatic illnesses, and (3) trauma and adverse behaviors in a Swedish nationwide sample. As sensitivity analyses, we examined the temporal order of these associations, sex-specific differences for individuals, and those with siblings diagnosed with BPD.

## Materials and methods

### Study population

The study population included individuals born in Sweden between January 1st, 1973 and December 31st, 1993 with a personal identity number and a biological mother identifiable in the register (2,177,075). We excluded individuals with a congenital malformation (113,566), those who died before age 18 (6972), and/or emigrated before age 18 (62,664). Thus, 1,969,839 individuals were included in our study.

### Data sources

Swedish personal identity numbers were used to link multiple Swedish registers in order to identify the cohort and create the analyzed variables, termed here as “indicators” (Table 1) [26–30]. The National Patient Register (NPR) contains administrative data from in-patient and specialist outpatient care (but not primary care) with diagnosis made by licensed medical doctors. The diagnoses have been externally validated by reviewing a random subset of patient’s medical records with comparison to the received diagnostic code. The positive predictive value of the psychiatric and somatic diagnoses in the register is high; out of the investigated medical records 80% or more retained the stated diagnostic code upon review [29]. Physical trauma requiring medical attention was found to have an acceptable positive predictive value of 74% [30, 31].

**Table 1:** Utilized Swedish national registers.

Source	Contained information	Use in study
National Patient Register	ICD diagnoses from all inpatient and outpatient specialist care after 2001; while only information on inpatient is available before this date. During our follow up period, two ICD revisions were used: the ICD-9 from 1987 until 1996 and the ICD-10 from January 1, 1997 and onwards	Determine all psychiatric and somatic illnesses
National Crime Register	All criminal convictions in the Swedish general court	Determine criminal convictions
Longitudinal Integration Database for Health Insurance and Labor Market Studies	Income, use of social services and benefits, and neighborhood quality	Determine poverty and neighborhood quality
Medical Birth Register	Information on birth, birthdate, and maternal prenatal period.	Identify cohort
Migration Register	Immigration and emigration to and from Sweden	Identify cohort and for censoring
Cause of Death Register	Date and cause of death	Identify cohort and for censoring

BPD diagnosis was defined by receiving an Emotionally Unstable Personality Disorder diagnosis in the NPR (ICD 10th revision: F60.3) by psychiatrists in in- or outpatient psychiatric clinics. In one validation study, structured interviews had been used in 36% of examined personality disorders (including other personality disorders than BPD), as identified from medical charts. However, the positive predictive value, i.e., proportion of diagnoses validated upon review, for the 26 BPD-diagnoses investigated was high regardless if structured interviews had been used or not, between 77% (based on DSM-criteria) and 100% (based on ICD-criteria); inter-rater agreement was between 85% (ICD-criteria) and 100% (DSM-criteria) [32]. Another validation study based on 70 medical charts with a BPD-diagnosis, and reported a positive predictive value of 81%, with an inter-rater agreement of 93% [33].

The indicators in our study were selected based on an existing data linkage, which contains a subset of all ICD-codes (Supplementary Table 1). Indicators were placed into three groups: psychiatric disorders, somatic illnesses, and trauma and adverse behaviors (Supplementary Tables 2–4). As a sensitivity analysis, we analyzed the three most common subcategories for umbrella indicators with many subtypes, e.g., autoimmune disorders. Adverse behaviors included violent/nonviolent crime and self-harm. Trauma included accidents requiring medical attention, violent crime victimization requiring medical attention, death of a close family member, and childhood neighborhood quality.

We only considered time of the first observed event for all indicators, except for childhood poverty and neighborhood quality.

The study was approved by the Regional Ethics Committee in Stockholm, Sweden (Dnr 2013/862 31/5). As our study participants were non-identifiable, no informed consent was needed by Swedish law.

### Statistical analysis

#### Cumulative incidence

First, we estimated cumulative incidence for those with and without a BPD diagnosis in order to quantify the associations between BPD and the indicators on an absolute scale. Each patient with BPD was matched with ten individuals not diagnosed with BPD on birth year and sex. Follow up for individuals with BPD began at the date of the first observed BPD diagnosis and continued until the end of follow-up, December 31, 2013, this date was also used for their matched non-exposed individuals. We calculated 5-year cumulative incidence, interpreted as the probability of the event occurring within 5 years, while accounting for censoring. We used Kaplan-Meier estimation to estimate the cumulative incidence as 1 minus the survival function.

#### Associations between BPD diagnosis and the indicators

To quantify the association between BPD and our indicators, hazard ratios (HR) with 95% confidence intervals (CI) were obtained using sex-stratified Cox regression. Age was used as an underlying time score and we adjusted for birth cohort (1973–1977, 1978–1982, 1983–1987, and 1988–1993). BPD diagnosis was treated as a binary, time-constant, exposure regardless of when the diagnosis occurred. We followed each individual from birth or the start of ICD-9, January 1, 1987, until the first instance of either death, emigration, indicator occurrence, or end of follow-up. We did not account for competing risks, since standard methods introduce changes in the association dependent on whether the exposure is associated with the competing outcome [34].

We adjusted the analysis for multiple testing according to Benjamini–Hochberg method, with an alpha of 0.05, we obtained a false discovery rate *p* value threshold of 8.34 · 10^−58^ for the main analysis and 0.04 for secondary analyses [35].

### Secondary analyses

#### Sex-separated and sibling analysis

To evaluate potential differences between males and females diagnosed with BPD, we repeated the cumulative incidence-, association-, and time-varying analyses by analyzing males and females separately. To investigate potential etiological differences, we repeated the association analyses among men and women separately, split by exposure being having a full sister or having a full brother with a BPD diagnosis.

#### Time-varying sensitivity analyses

By not considering timing of exposure in our main analysis, we assumed that individuals were exposed since birth although the BPD diagnosis occurred at a later age. This means that we were “borrowing information from the future”, an approach that may introduce bias. Therefore, we repeated all Cox regression analyses comparing the indicators before or after a BPD diagnosis to treat exposures as time-varying. Risk factor analyses considered the indicators to be exposures prior to a BPD diagnosis, while outcome analyses considered indicators as an outcome following a BPD diagnosis.

SAS was used for data management and all subsequent analysis was done in R using the survival package [36].

## Results

### Descriptive statistics

The cohort consisted of 1,969,839 individuals (48.8% female) with 12,175 individuals with BPD (85.3% female; 0.6% of sample) (Table 2; absolute values Supplementary Tables 5–7). Total follow-up time was 32,637,932 person-years, calculated from the first possible time of BPD diagnosis, i.e., from its introduction in 1997, and onward. The diagnosis was evenly distributed between birth year cohorts, and the total cohort had a mean age of 29.69 years at the end of follow-up.

**Table 2:** Descriptive information.

	Total cohort No. %		With BPD diagnosis No. %		Without BPD diagnosis No. %	
Individuals	1,969,839	100%	12,175	0.6%	1,957,664	99.4%
Follow-up time, person-years	32,637,932		202,513		32,435,419	
Males	1,007,774	51.2%	1,785	14.7%	1,005,989	51.4%
Females	962,065	48.8%	10,390	85.3%	951,675	48.6%
Birth year						
1973–1977	461,587	23.4%	2,356	19.4%	459,231	23.4%
1978–1982	421,516	21.4%	2,978	24.5%	418,538	21.3%
1983–1987	439,982	22.3%	3,487	28.6%	436,495	22.2%
1988–1993	646,754	32.8%	3,354	27.5%	643,400	32.9%

#### Cumulative incidences

**Fig. 1:**
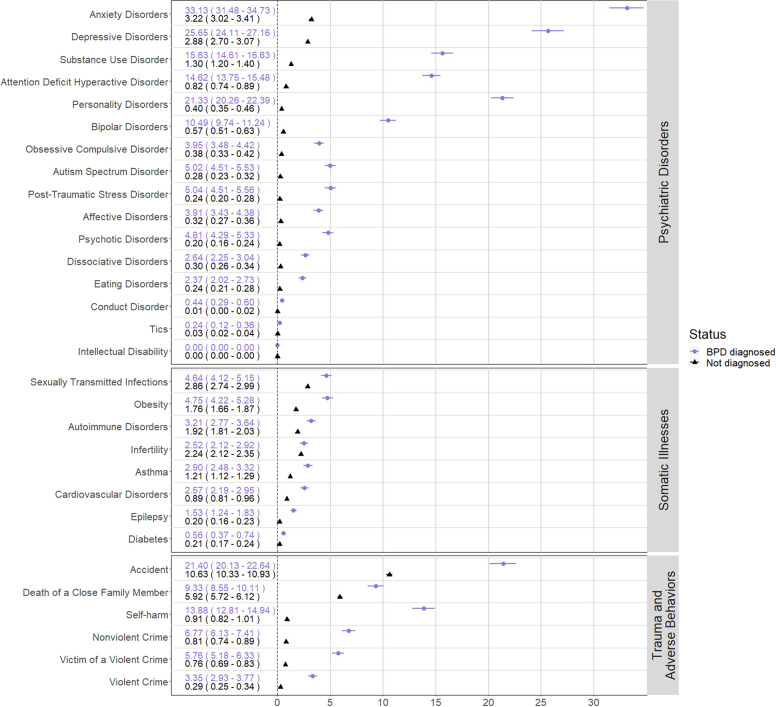
Cumulative incidence by 5 years after Borderline Personality Disorder diagnosis, estimate in percent and (95% confidence interval). The cumulative incidence of each of the main indicators broken down by subgroups.

The 5-year cumulative incidence showed increased incidences in individuals with BPD compared to the matched control sample for all indicators, except intellectual disability (Fig. 1; Supplementary Figs. 1–7). The largest cumulative incidences were for anxiety disorders (Cumulative incidence [95% CI]; BPD 33.13% [31.48–34.73%]; not BPD (NBPD) 3.17% [2.98–3.79%]), major depressive disorder (BPD 25.65% [24.11–27.16%]; NBPD 3.04% [2.85–3.24%]) personality disorders (BPD 21.33% [20.26–22.39%]; NBPD 0.36% [0.31–0.41%]), accidents requiring medical attention (BPD 21.50% [20.13–22.64%]; NBPD 10.91% [10.60–11.21%]), and attention-deficit hyperactive disorder (BPD 14.62% [13.75–15.48%]; NBPD 0.81% [0.74–0.88%]). Intellectual disability had zero first event occurrences after date of BPD-diagnosis for both exposed and unexposed.

#### Associations between BPD diagnosis and indicators

**Fig. 2:**
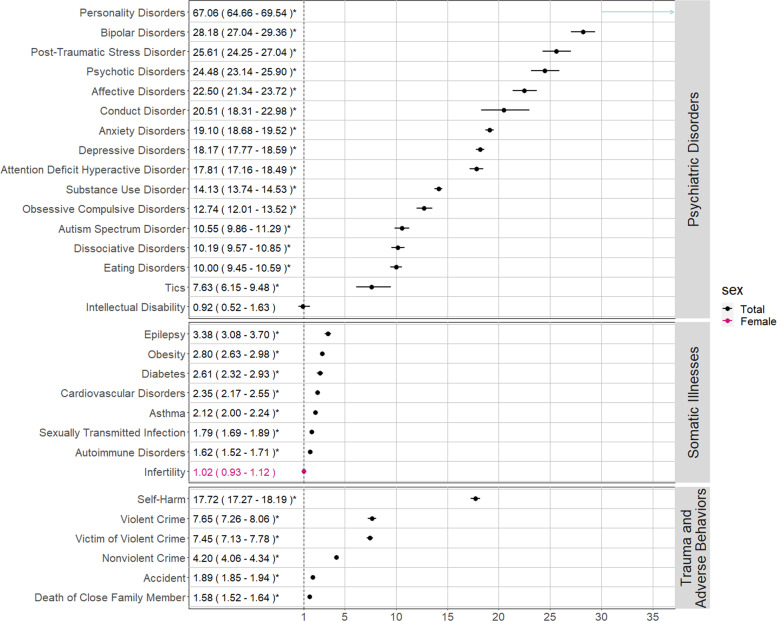
Associations with Borderline Personality Disorder diagnosis, hazard ratio and (95% confidence interval). *Statistically significant after correcting for multiple testing using the Benjamini-Hochberg method, resulting in a *p* value threshold of 8.34 · 10^−58^.

All HRs for BPD and the indicators included in our main analysis were statistically significant, i.e., larger than 1, after correcting for multiple testing, except for intellectual disability and infertility in females (Fig. 2). The majority of the indicators under the umbrella categories were also statistically significantly larger than 1 (Supplementary Figs. 8 and 9).

##### Psychiatric disorders

Psychiatric disorders had the highest HRs across all analyses. The highest HRs were personality disorders not including BPD (HR [95% CI] 67.06 [64.66–69.54]), bipolar disorders (28.18 [27.04–29.36]), and PTSD (25.61 [24.25–27.04]). Psychotic disorders were also elevated (24.48 [23.14–25.90]).

##### Somatic illnesses

The largest HRs for the association between BPD and somatic illnesses were for epilepsy (3.38 [3.08–3.70]), obesity (2.8 [2.63–2.98]), and diabetes (2.61 [2.32–2.93]).

##### Traumatic events and adverse behaviors

The strongest association regarding traumatic events was violent crime victimization (7.45 [7.13–7.78]). Death of a close family member also had a positive association (1.58 [1.52–1.64]). Self-harm had the highest hazard ratios of adverse behaviors (17.72 [17.27–18.19]). Committing a violent crime (7.65 [7.26–8.06]) had a stronger association than nonviolent crimes (4.20 [4.06–4.34]). Impulsive nonviolent crimes, i.e., property damage (6.66 [6.05–7.33]), had a stronger association than planned crime, possessing fake identification (1.94 [1.19–3.17]). Although they were analyzed as risk factors by definition, the HRs for poverty in childhood (1.93 [1.86–2.00]) and childhood neighborhood quality (1.52 [1.47–1.59]) were positively associated with BPD diagnosis (Supplementary Fig. 10 and Supplementary Table 5).

### Secondary analyses

#### Sex-separated and sibling analysis

Females with BPD had higher cumulative incidences compared to males with BPD in nearly all somatic disorders, e.g., obesity (male 1.80 [0.97–2.62]; female 5.29 [4.68–5.90]); while the inverse was true for adverse behaviors and traumas, e.g., committing a violent crime (male 10.27 [8.14–12.35]; female 2.44 [2.06–2.83]). However, the CIs frequently overlapped between the sexes (Supplementary Figs. 11–23). HRs were largely uniform (Supplementary Fig. 24). Males had higher HRs for bipolar disorder (male 36.31 [32.62–40.41]; female 27.11 [25.93–28.33]), PTSD (male 34.99 [29.54–41.44], female 24.72 [23.35–26.18]) and affective disorders (male 31.42 [27.63–35.72]; female 21.32 [20.13–22.59]). Somatic disorders were mostly uniform across sex. Committing a violent crime was relatively more elevated in females (male 6.90 [6.38–7.46]; female 8.25 [7.68–8.86]). Additionally, being a victim of a violent crime requiring medical attention had a higher HR in females (male 5.05 [4.56–5.58]; female 8.58 [8.17–9.01]).

Individuals with siblings diagnosed with BPD had increased rates of indicators, however, many CIs contained 1, especially within somatic disorders (Supplementary Tables 9–11). Individuals with brothers diagnosed with BPD had higher HRs than those with sisters who were diagnosed in 87 out of 123 indicators. Psychiatric disorders had the strongest association, e.g., PTSD (males with brothers diagnosed with BPD (BBPD) 6.76 [3.74–12.24], males with sisters diagnosed with BPD (SBPD) 2.62 [1.78–3.87], females with BBPD 4.09 [2.72–6.16], females with SBPD 3.62 [3.02–4.35]).

#### Time-varying sensitivity analyses

Broadly, the magnitude of HRs for each indicator stayed relatively consistent when treating indicators as a risk factor prior to, or as an outcome following, a BPD diagnosis (Supplementary Tables 8, 12–13 and Supplementary Figs. 10, 25, and 26. Some notable exceptions were personality disorders (HR [95% CI] risk factor for subsequent BPD diagnosis 71.50 [68.36–74.78]; outcome following a BPD diagnosis 43.77 [41.40–46.28]), bipolar disorders (risk 35.94 [34.16–37.82]; outcome 17.27 [16.12–18.51), and psychotic disorders (risk 25.82 [24.19–27.56]; outcome 17.96 [16.23–19.87]) which had higher HRs leading up to a BPD diagnosis, and epilepsy (risk 2.89 [2.59–3.22]; outcome 5.36 [4.53–6.34]) that had a higher HR following a BPD diagnosis.

## Discussion

In this population-based study, which included 12,175 individuals with BPD in a total sample of 1,969,839 Swedes, we found that BPD was associated with an increased risk for psychiatric comorbidities, somatic illnesses, traumatic events, and adverse behaviors. Our findings replicate previously known associations and identify unknown or understudied associations, e.g., epilepsy, infertility, and death of close family members. Moreover, this work extends findings to include both sexes and their siblings.

### Psychiatric disorders

BPD was strongly associated with all psychiatric disorders except intellectual disability. We found a robust association between BPD and all other personality disorders, mood disorders, eating disorders, PTSD, and substance use disorders. Certain findings need additional vigilance from clinicians and researchers, e.g., the strong association with psychotic disorders. This finding follows the historical implication of the term borderline, coined to indicate that the patients were on the borderline between psychosis and neurosis [37]. Psychotic experiences are often treated as transient symptoms according to DSM guidelines, although symptoms are often perpetual [12]. Our finding highlights the importance of carefully assessing psychotic symptoms in BPD, and indicates that psychotic disorders are indeed overrepresented in individuals with BPD [38].

### Somatic illnesses

Individuals with BPD had a higher risk of almost all somatic comorbidities in the main analysis except for female infertility. A Danish study also found a positive association between somatic disorders and combined personality disorders, however, their estimates were smaller than our results [39]. This could suggest that a BPD diagnosis has a worse prognosis than other personality disorders. In line with the literature, epilepsy and metabolic-related comorbidities, such as type 2 diabetes and obesity, had the strongest associations [10]. Previous studies have indicated a relationship between epilepsy and BPD, and here we show a clear association [40].

### Trauma and adverse behaviors

Individuals with BPD were at a higher risk of all traumatic events both before and after receiving their diagnosis. Namely, individuals with BPD were at a higher risk of seeking medical care due to violent crime victimization, especially sexual assault, which had the strongest association. This supports the expansive literature linking sexual assault and a BPD diagnosis [19]. Second, there was a positive association between BPD and the death of a close family member, which has only been reported in studies involving the death of a parent in childhood [41]. Third, our study identified an underreported positive link between childhood poverty and poor neighborhood quality and subsequent BPD diagnosis [42]. Although the CIs overlapped, sex-separated analysis found that males had higher rates of traumatic events, except for sexual assault, fitting within previous literature on BPD symptoms and trauma [43].

The consistent time-varying results provide evidence for the theory of a close, cyclical relationship between trauma and BPD [1, 20]. Our sibling analysis found evidence to support an overlap in genetic and/or environmental etiology between traumatic events and BPD diagnosis, previously theorized to be present [44].

As expected, BPD individuals had higher instances of adverse behaviors: self-harm, violent and nonviolent crime. In line with previous findings, the most common criminal behaviors were aggressive in nature, such as making violent threats and assault [2, 4]. Additionally, our study identified that impulsive nonviolent crimes such as property damage or petty theft are more common than planned crimes, e.g., having fake identification.

### Sex differences and time-varying findings

The HRs were largely uniform across sexes, even though the absolute proportions, i.e., cumulative incidences, sometimes differed substantially. This suggests that this disorder confers similar increase in rates of comorbidities between males and females. A diagnostic bias between the sexes could result in an inflated estimate for males with BPD, as males who receive the correct diagnosis might have a more severe symptom presentation.

Individuals with a sibling with BPD had higher rates of psychiatric disorders, trauma, and adverse behaviors but not somatic disorders. HRs were higher in individuals with a brother diagnosed with BPD compared to those with a sister diagnosed for the majority of indicators. Families with a male diagnosed with BPD appear to have a more severe phenotype and vulnerability to psychiatric disorders [45]. However, this must be interpreted with caution as the CIs overlapped for having a brother or sister diagnosed with BPD. Follow-up on these associations is needed.

As the associations for the time-varying analyses were largely consistent with the main analysis, ignoring time of BPD diagnosis did not introduce bias that invalidated our inferences.

### Strengths, weaknesses

In the largest and most detailed BPD study to date, we were able to capture all Sweden-born individuals with BPD with prospective follow-up using national records. This considerable sample size allowed us to thoroughly examine a variety of understudied variables in a representative sample.

However, this study comes with caveats. First, although the NPR and BPD diagnostic codes are well-validated, the extent to which these comorbidities may be misdiagnosed is unclear [29, 32, 33]. However, one may argue that diagnoses, if not correctly diagnosed, may reflect levels of symptom presentation even if the full criteria of diagnosis is not met. Further, the prevalence of a BPD diagnosis in our sample (0.6%) is conservative compared to the projected median estimate of 1.7% [1]. This likely indicates that many individuals who meet the criteria for a BPD diagnosis remain undiagnosed. And, as a BPD diagnosis generally takes multiple, intensive clinical interviews, it is likely that we are capturing individuals with more severe BPD symptoms.

Next, we were only able to identify the instances of the indicators identifiable in the register, this means the true estimate of these incidences may be higher in under-reported or less severe cases. Some individuals with BPD may be less willing to seek certain types of medical care due to distrust or stigmatization from the health care system, which may attenuate our results [46]. Additionally, trauma is both an objective and deeply subjective experience; and we are unable to capture the subjective experience [21]. Finally, symptoms of BPD, like all psychiatric disorders, are continuously distributed across the population rather than a binary diagnosis. We are unable to examine specific BPD symptoms which limits the information gained from this study.

### Future directions for research

This study identifies many avenues for needed research. The comorbidities between BPD and psychotic disorders should be examined further. Moreover, the association with death of a close family member opens the question of the heritability of premature mortality in families [47]. Also, studies should examine these associations in other severe psychiatric disorders, such as bipolar disorder, to determine what associations are specific to BPD rather than general to severe psychiatric disorders [48, 49]. Finally, further research should carefully expand on any causal assumptions about the nature of BPD.

### Clinical implications

Our results indicate individuals with BPD frequently use health care services for a variety of psychiatric and somatic conditions. Unfortunately, despite their needs, the interpersonal difficulties and stigma surrounding individuals with BPD can be challenging for clinicians, which often results in poor care or premature ending of treatment [46, 50]. Thus, there is a clear need for support and advocates for individuals with BPD navigating the health care system. Clinicians should be aware of these difficulties within the health care system and offer adapted health education or referrals, e.g., a dietitian to prevent type 2 diabetes. Additionally, implementing already developed anti-stigmatization methods could help improve the doctor–patient relationship [50, 51].

## Conclusions

BPD was associated with nearly all of the more than 30 indicators of psychiatric disorders, somatic illnesses, trauma, and adverse behavior. The associations were consistent across sex and temporality. This paper can serve as an atlas for associations within the aforementioned categories, many of which have previously been un- or under-reported, and can lead the way towards further causal and etiological research. Critically, the clinical implications indicate that increased support is needed for patients surrounding health care visits. It is hopeful that this provides the groundwork towards an understanding and increased awareness for this patient group.

## Supplementary information

Supplementary Material
