# Supplementary material for: Borderline personality disorder: associations with psychiatric disorders, somatic illnesses, trauma, and adverse behaviors
Source: Mol Psychiatry. 2022 Mar 18;27(5):2514–21. doi: 10.1038/s41380-022-01503-z (PMC9135625; doi:10.1038/s41380-022-01503-z)
Supplement: Supplementary file 1 — Supplementary Material [file 41380_2022_1503_MOESM1_ESM.docx]

Borderline Personality Disorder: associations with psychiatric disorders, somatic illnesses, trauma, and adverse behaviors

Supplemental Material

Ashley E. Tate^1^, Hanna Sahlin^2^, Shengxin Liu^1^, Yi Lu^1^, Sebastian Lundström^3,4^, Henrik Larsson^1,5^, Paul Lichtenstein^1^, Ralf Kuja-Halkola^1^

Author Affiliations:

1. Department of Medical Epidemiology and Biostatistics, Karolinska Institutet, Solna, Sweden
2. Centre for Psychiatry Research, Department of Clinical Neuroscience, Karolinska Institutet, Stockholm, Sweden
3. Centre for Ethics, Law and Mental Health (CELAM), University of Gothenburg, Gothenburg, Sweden
4. Gillberg Neuropsychiatry Centre, University of Gothenburg, Gothenburg, Sweden
5. School of Medical Sciences, Örebro University, Örebro, Sweden

**Supplementary Table 1. Ordered diagnoses from the Swedish National Patient Register**

| **ICD categories** | **ICD-9** | **ICD-10** |
| --- | --- | --- |
| Certain infectious and parasitic diseases | 001-139 | A00-B99 |
| Certain disorders involving the immune mechanism | 279 | D80-D89 |
| Endocrine, nutritional and metabolic diseases | 240-246, 250-259, 278 | E00-E35, E65-E68 |
| Psychiatric disorders and behavioral disorders | 290-319 | F00-F99 |
| Diseases of the nervous system | 320-349, 356-359 | G00-G47, G60-G73, G91 |
| Conjunctivitis | 372 | H10 |
| Diseases of the circulatory system | 390-448, 456 | I00-I79, I98.3 |
| Respiratory Diseases | 460-466, 470-478, 480-487, 490-496, 514 | J00-J46 |
| Diseases of the digestive system | 530-535, 540-542, 555-558, 570-577, 579 | K25-K37, K50-K51, K70-K85, K90 |
| Diseases of the skin and subcutaneous tissue | 680-698, 701, 708-709 | L00-L54, L90-L95 |
| Diseases of the musculoskeletal system and connective tissue | 710-721, 725-729 | M00-M68 |
| Diseases of the genitourinary system | 580-583, 590, 592, 594-595, 601-608, 628 | N00-N08, N10-N12, N20-N21, N30, N41-N49, N97 |
| Pregnancy, childbirth, and the puerperium | 642, 648, 670 | O10-O16, O20-26, O85-86 |
| Certain conditions originating in the perinatal period | 760-779 | P00-P96 |
| Congenital malformation, deformations, and chromosomal abnormalities | 740-759 | Q00-Q99 |
| Symptoms, signs and abnormal clinical and laboratory findings, not elsewhere classified | 784, 786 | R05-R06, R47-R49 |
| Injury, poisoning and certain other consequences of external causes | 800-995 | S00-T98 |
| External causes of morbidity and mortality | E807-E999 | V01-Y98 |

**Supplementary Table 2. ICD codes used to derive psychiatric disorders and subcategories**

| **Indicators** | **Subcategories** | **ICD-9** | **ICD-10** |
| --- | --- | --- | --- |
| Affective disorders |  | 300E, 300X, 301B | F34 – F39 |
| Anxiety |  | 300A, 300C | F40, F41, F48 |
| Attention deficit hyperactive disorder |  | 314 | F90 |
| Autism spectrum disorder |  | 299A | F84 |
| Bipolar disorders |  | 296A-296E, 296W, 296X | F30, F31 |
| Conduct disorder |  | 312 | F91 |
| Depression |  | 296B, 311 | F32, F33 |
| Dissociative disorders |  | 298B, 298C, 298W, 298X, 300B, 300F-X, 301W, 306A-X, 307W, 307X, 784G, V40X | F44, F45 |
| Eating Disorders |  | 307B 307F | F50.0, F50.1, F50.2, F50.3, & F50.9 |
| Intellectual disability |  | 317 – 319 | F70 - F79 |
| Obsessive compulsive disorder |  | 300D | F42 |
| Personality disorders |  |  | F60 (not F603) |
|  | Antisocial personality disorder | 301G | F602 |
|  | Avoidant personality disorder | 301H | F606 |
|  | Personality disorder not specified | 301I | F609 |
| Post-traumatic stress disorder |  | 308, 309 | F431 |
| Psychotic disorders |  | 295A-295E, 295G, 295W, 295X, 295H | F20, F21, F25 |
|  | Schizophrenia | 295A-295E, 295G, 295W, 295X | F20 |
|  | Schizoaffective disorder | F295H | F25 |
|  | Schizotypal disorder ^a^ | 295E | F21 |
| Substance Misuse |  | 291A-X, 292A-X, 294A, 303, 304A-X, 305A-X | F10, F11-F19 (not F17) |
| Tic disorder |  | 307C | F95.0 – F95.2, F59.9 |

^a^ Although considered a personality disorder in the DSM-5, schizotypal disorder is considered a psychotic disorder in the ICD-10

**Supplementary Table 3. ICD codes used to derive somatic illnesses and subcategories**

| **Indicators** | **Subcategories** | **ICD-9** | **ICD-10** |
| --- | --- | --- | --- |
| Asthma |  | 493A-X | J45‐J46 |
| Autoimmune disorders ^1^ |  | 704A, 258B, 136B, 694F, 579A, 555, 710D, 242A, 357.A, 245.C, 287.A, 287.D, 580-582, 446.1, 710.W, 340, 358.A, 694.E, 725, 446.0, 571.F, 696, 390-391, 392, 714.A, 034.1, 710A-C, 446F-G, 556, 709.A, 446.E | G04, L63, G13.1, D68.61, E31.0, M35.2, L12.0, K90.0, M30.1, K50, M33.90, E05.0, G61.0, E06.3, D69.0, D69.3, N00-01, N03, N05, M30.3, M31.7, M35.1, G35, G70.0, L10.0, M35.3, M30.0, K74.3, L40, I00-02, M06, A38.9, M34, M35.0, M32, M31.5, M31.1, E10.9, K51, L80, M31.3 |
|  | Intestinal malabsorption | 5790 | K90 |
|  | Psoriasis | 683 | L40 |
|  | Ulcerative colitis | 556 | K51 |
| Cardiovascular disorders |  | 397X, 394A-B, 394C, 394X, 395A-C, 395X, 396X, 397A-B, 397X, 398A, 398X, 410A-B, 410W-X, 411A-C, 411X, 412X, 413X, 414A-B, 414W, 414X, 420X, 421A, 421X, 422X, 423A-C, 423W, 423X, 424A-D, 424X, 425A-B, 425D-F, 425X, 426A-H, 426W, 426X, 429A-G, 429W, 429X | I05 – I109, I20 – I28, I30 - 152 |
|  | Cardiac arrhythmias | 426H | I499 |
|  | Paroxysmal tachycardia | 427X | I471 |
|  | Rheumatic heart diseases | 401 | I109 |
| Diabetes |  | 250 | E10, E11 |
|  | Type 1 |  | E10 |
|  | Type 2 |  | E11 |
| Epilepsy |  | 345J-345N, 345P, 345Q, 345W, 345X | G40 |
| Infertility |  | 628 | N97 |
| Obesity |  | 278 | E66, Z71.3 |
| Sexually transmitted infection |  | 090A – 099X, 279G, 279J, 279K | A50 – A64, B20, B373 |
|  | Chlamydia | 0998 | A56 |
|  | Herpes viral infection | 0541 | A60 |
|  | Venereal warts | 0913 | A630 |

^1^ Autoimmune ICD codes were obtained from Mataix-Cols D, et al., (2018) (1)

**Supplementary Table 4. ICD codes used to derive trauma and adverse behaviors and subcategories**

| Indicators | Subcategories | Source | Calculation |
| --- | --- | --- | --- |
| Accident |  | National Patient Register | ICD 10: W, X00 – X58  ICD 9: 810 – 949, 99 |
|  | Fall | National Patient Register | ICD 10: W0 – W1  ICD 9: 88E |
|  | Object enters eye | National Patient Register | ICD 10: W44  ICD 9: 915E |
|  | Struck by object | National Patient Register | ICD 10: W22  ICD 9: E917 |
| Death of a close family member |  | Cause of Death Register | Family connections identified through the Multi-generation register |
|  | Death of father | Cause of Death Register | Family connections identified through the Multi-generation register |
|  | Death of mother | Cause of Death Register | Family connections identified through the Multi-generation register |
|  | Death of sibling | Cause of Death Register | Family connections identified through the Multi-generation register |
| Neighborhood deprivation ^1^ |  | Longitudinal Integration Database for Health Insurance and Labor Market Studies (LISA) | Created from the highest quartile of the neighborhood deprivation score by either averaging the values from ages 5 to 10 or the value at the start of LISA which was used to measure income (year 1990) whichever came last |
| Poverty |  | LISA | Created from the lowest quartile of family disposable income by either averaging the values from ages 5 to 10 or the value at the start of LISA which was used to measure income (year 1990) whichever came last |
| Nonviolent crime |  | National Crime Register | Identified through Sweden-specific criminal codes |
|  | Petty theft | National Crime Register | Identified through Sweden-specific criminal codes |
|  | Fake passports or identification | National Crime Register | Identified through Sweden-specific criminal codes |
|  | Property damage | National Crime Register | Identified through Sweden-specific criminal codes |
| Self-harm |  | National Patient Register | ICD 10: X60 – X84, Y10 – Y34  ICD 9: 95, 98 |
| Victim of violent crime requiring medical attention |  | National Patient Register | ICD 10: X85-Y09  ICD 9: 96 |
|  | Physical Assault | National Patient Register | ICD 10:Y04  ICD 9: 960E |
|  | Sexual Assault | National Patient Register | ICD 10: Y05  ICD 9: 961E |
|  | Abuse | National Patient Register | ICD 10: Y07  ICD 9: 967E |
| Violent crime |  | National Crime Register | Identified through Sweden-specific criminal codes |
|  | Assault | National Crime Register | Identified through Sweden-specific criminal codes |
|  | Threats of violence | National Crime Register | Identified through Sweden-specific criminal codes |
|  | Committing bodily injury | National Crime Register | Identified through Sweden-specific criminal codes |

^1^ Score originally created by Sariaslan, et. al., (2015) (2)

**Supplementary Table 5. Absolute number of psychiatric disorder diagnoses**

| **Indicators** | **Total No. %** | | **Diagnosed with BPD No. %** | | **Not Diagnosed with BPD No. %** | |
| --- | --- | --- | --- | --- | --- | --- |
| Affective disorders | 13 265 | 0.7% | 1 62 | 14.5% | 11 303 | 0.6% |
| Anxiety | 122 538 | 6.2% | 10 184 | 75.4% | 112 354 | 5.7% |
| Attention deficit hyperactive disorder | 43 310 | 2.2% | 3 638 | 26.9% | 39 672 | 2.0% |
| Autism spectrum disorder | 19 779 | 1.0% | 1 108 | 8.2% | 18 671 | 1.0% |
| Bipolar disorders | 18 603 | 0.9% | 3 267 | 24.2% | 15 336 | 0.8% |
| Conduct disorder | 4 135 | 0.2% | 407 | 3.0% | 3 728 | 0.2% |
| Depression | 113 685 | 5.8% | 9 394 | 69.6% | 104 291 | 5.3% |
| Dissociative disorders | 16 992 | 0.9% | 1 478 | 10.9% | 15 514 | 0.8% |
| Eating Disorders | 15 496 | 0.8% | 1 532 | 11.3% | 13 964 | 0.7% |
| Intellectual disability | 2 277 | 0.1% | 13 | 0.1% | 2 264 | 0.1% |
| Obsessive compulsive disorder | 15 455 | 0.8% | 1 399 | 10.4% | 14 056 | 0.7% |
| Personality disorders (PD) | 19 889 | 1.0% | 5 667 | 42.0% | 14 222 | 0.7% |
| PD: Antisocial personality disorder | 1 246 | 0.1% | 328 | 2.7% | 918 | 0.0% |
| PD: Avoidant personality disorder | 1 683 | 0.1% | 278 | 2.3% | 1 405 | 0.1% |
| PD: Not specified personality disorder | 12 657 | 0.6% | 3 779 | 30.8% | 8878 | 0.5% |
| Post-traumatic stress disorder | 10 326 | 0.5% | 1 961 | 14.5% | 8 365 | 0.4% |
| Psychotic disorders | 32 693 | 1.6% | 5 577 | 34.2% | 27 116 | 1.4% |
| Psychotic: Schizophrenia | 10 959 | 0.6% | 1 950 | 12.0% | 9 009 | 0.5% |
| Psychotic: Schizoaffective disorder | 20 715 | 1.0% | 4 382 | 26.9% | 16 333 | 0.8% |
| Psychotic: Schizotypal disorder | 1 802 | 0.1% | 793 | 4.9% | 1 009 | 0.1% |
| Substance Misuse | 90 577 | 4.6% | 6 218 | 46.1% | 84 359 | 4.3% |
| Tic disorder | 3 232 | 0.2% | 87 | 0.7% | 3 145 | 0.2% |

**Supplementary Table 6. Absolute number of somatic illness diagnoses**

| **Indicators** | **Total No. %** | | **Diagnosed with BPD No. %** | | **Not Diagnosed with BPD No. %** | |
| --- | --- | --- | --- | --- | --- | --- |
| Asthma | 101 682 | 5.2% | 1 302 | 10.7% | 100 380 | 5.1% |
| Autoimmune disorders ^a^ | 103 814 | 5.3% | 1 134 | 9.3% | 102 680 | 5.2% |
| Autoimmune: Intestinal malabsorption | 14 357 | 0.7% | 168 | 1.4% | 14 189 | 0.7% |
| Autoimmune: Psoriasis | 21 816 | 1.1% | 238 | 1.9% | 21 578 | 1.1% |
| Autoimmune: Ulcerative collitis | 14 398 | 0.7% | 101 | 0.8% | 14 297 | 0.7% |
| Cardiovascular disorders (CVD) | 46 350 | 2.4% | 598 | 4.9% | 45 752 | 2.3% |
| CVD: cardiac arrhythmias | 3 238 | 0.2% | 38 | 0.3% | 3 200 | 0.2% |
| CVD: Paroxysmal tachycardia | 5 065 | 0.3% | 53 | 0.4% | 5 012 | 0.3% |
| CVD: Rheumatic heart diseases | 10 852 | 0.6% | 141 | 1.1% | 10 711 | 0.5% |
| Diabetes | 19 593 | 1.0% | 289 | 2.4% | 19 304 | 1.0% |
| Diabetes: Type I | 16 900 | 0.9% | 199 | 1.6% | 16 701 | 0.9% |
| Diabetes: Type II | 5 809 | 0.3% | 166 | 1.4% | 5 643 | 0.3% |
| Epilepsy | 23 248 | 1.2% | 479 | 3.9% | 22 769 | 1.2% |
| Infertility | 41 447 | 2.1% | 453 | 3.7% | 40 994 | 2.1% |
| Obesity | 42 965 | 2.2% | 995 | 8.2% | 41 970 | 2.1% |
| Sexually transmitted infection (STI) | 101 645 | 5.2% | 1 335 | 11.0% | 100 310 | 5.1% |
| STI: Chlamydia | 26 163 | 1.3% | 313 | 2.5% | 25 850 | 1.3% |
| STI: Herpes viral infection | 24 877 | 1.3% | 449 | 3.7% | 24 428 | 1.2% |
| STI: Veneral warts | 52 997 | 2.7% | 595 | 4.8% | 52 402 | 2.7% |

| Indicators | Total No. % | | Diagnosed with BPD No. % | | Not Diagnosed with BPD No % | |
| --- | --- | --- | --- | --- | --- | --- |
| Accident | 787 952 | 39.9% | 6 895 | 56.4% | 781 057 | 39.8% |
| Accident: Fall | 500 290 | 25.4% | 4 506 | 36.7% | 495 784 | 25.3% |
| Accident: Object enters eye | 34 431 | 1.7% | 284 | 2.3% | 34 147 | 1.7% |
| Accident: Struck by object | 70 457 | 3.6% | 618 | 5.0% | 69 839 | 3.6% |
| Death of a close family member | 318 391 | 16.1% | 2 937 | 24.0% | 315 454 | 16.1% |
| Death of father | 183 788 | 9.3% | 1 716 | 14.0% | 182 072 | 9.3% |
| Death of mother | 89 562 | 4.5% | 899 | 7.4% | 88 663 | 4.5% |
| Death of sibling | 83 185 | 4.2% | 817 | 6.7% | 82 368 | 4.2% |
| Neighborhood deprivation ^1^ | 483 080 | 25.0% | 3 961 | 33.7% | 479 119 | 24.9% |
| Poverty ^2^ | 483 081 | 25.0% | 4 652 | 39.6% | 478 429 | 24.9% |
| Nonviolent crime (NV) | 199 925 | 10.1% | 3 489 | 28.7% | 196 436 | 10.0% |
| NV: Petty theft | 76 212 | 3.9% | 1 954 | 16.0% | 74 258 | 3.8% |
| NV: Fake passports or identification | 11 922 | 0.6% | 103 | 0.8% | 11 819 | 0.6% |
| NV: Property damage | 40 661 | 2.1% | 775 | 6.4% | 39 886 | 2.0% |
| Self-harm | 80 697 | 4.1% | 6 561 | 53.6% | 74 136 | 3.8% |
| Victim of violent crime requiring medical attention (VVC) | 68 829 | 3.5% | 2 135 | 17.5% | 66 694 | 3.4% |
| VVC: Physical Assault | 28 837 | 1.5% | 528 | 4.3% | 28 309 | 1.4% |
| VVC: Sexual Assault | 6 424 | 0.3% | 742 | 6.0% | 5 682 | 0.3% |
| VVC: Abuse | 29 838 | 1.5% | 859 | 7.0% | 28 979 | 1.5% |
| Violent crime (VC) | 68 889 | 3.5% | 1 473 | 12.0% | 67 416 | 3.4% |
| VC: Assault | 9 758 | 0.5% | 268 | 2.2% | 9 490 | 0.5% |
| VC: Threats of violence | 16 796 | 0.9% | 565 | 4.6% | 16 231 | 0.8% |
| VC: Committing bodily injury | 53 417 | 2.7% | 1 017 | 8.4% | 52 400 | 2.7% |

**Supplementary Table 7. Absolute number of instances of trauma and adverse behaviors**

^1^ Created from the lowest quartile of family disposable income by either averaging the values from ages 5 to 10 or the value at the start of the register Longitudinal Integration Database for Health Insurance and Labor Market Studies was used to measure income (year 1990) whichever came last

^2^ Created from the highest quartile of the neighborhood deprivation score by either averaging the values from ages 5 to 10 or the value at the start of the register Longitudinal Integration Database for Health Insurance and Labor Market Studies which was used to measure income (year 1990) whichever came last

**Supplementary Figure 1. Cumulative incidence of subcategories for 5 years after Borderline Personality Disorder diagnosis, estimate and (95% confidence interval) ^1^**


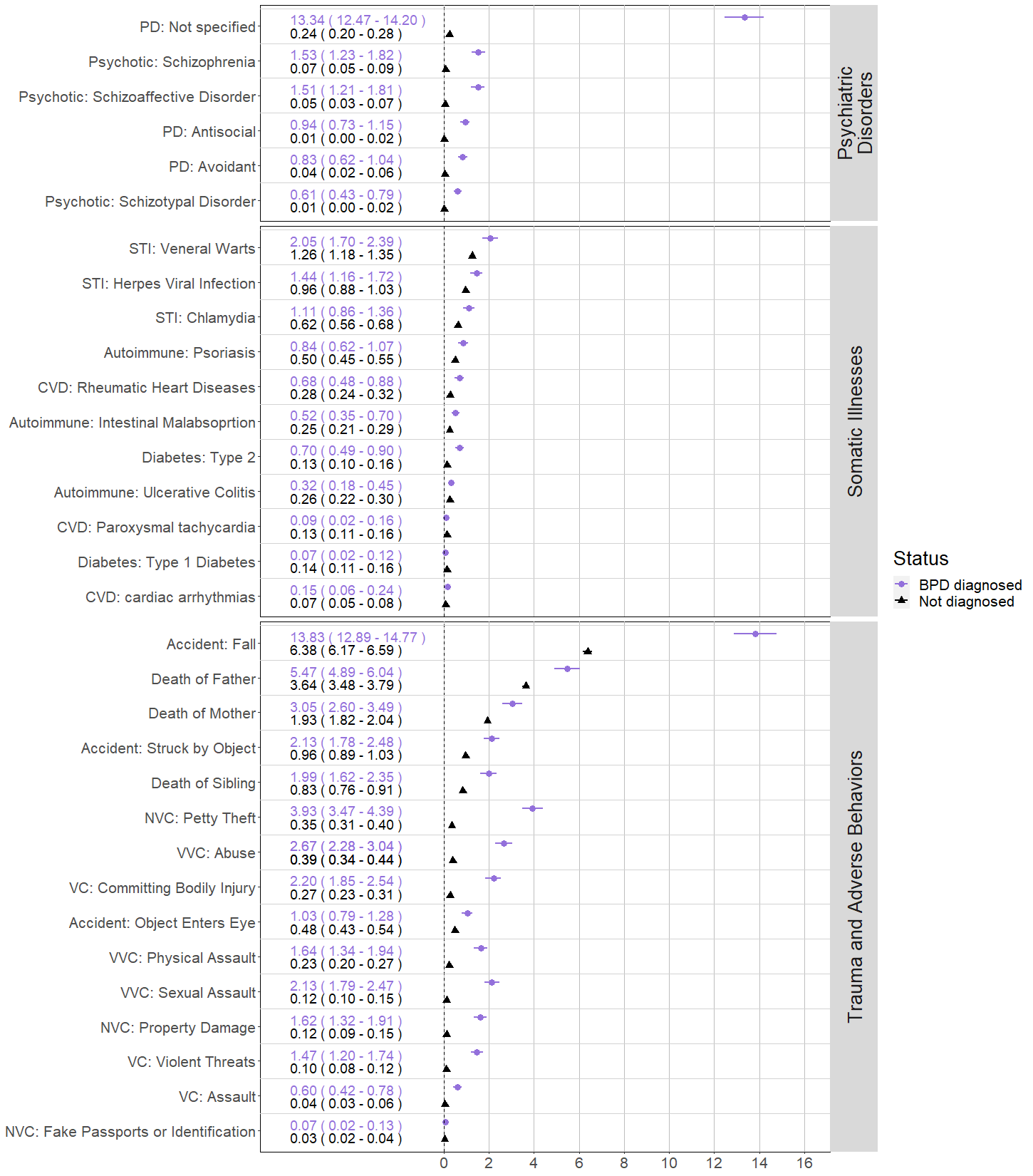


^1^ Abbreviations: Personality disorders (PD), Cardiovascular disorders (CVD), Sexually transmitted infection (STI), Victim of a violent crime requiring medical attention (VVC), Violent crime conviction (VC), Nonviolent crime conviction (NVC)

**Supplementary Figure 2. Cumulative incidence of psychiatric disorders with the highest values during 17 years following a BPD diagnosis in the total sample, estimates and 95% confidence intervals (shaded)**


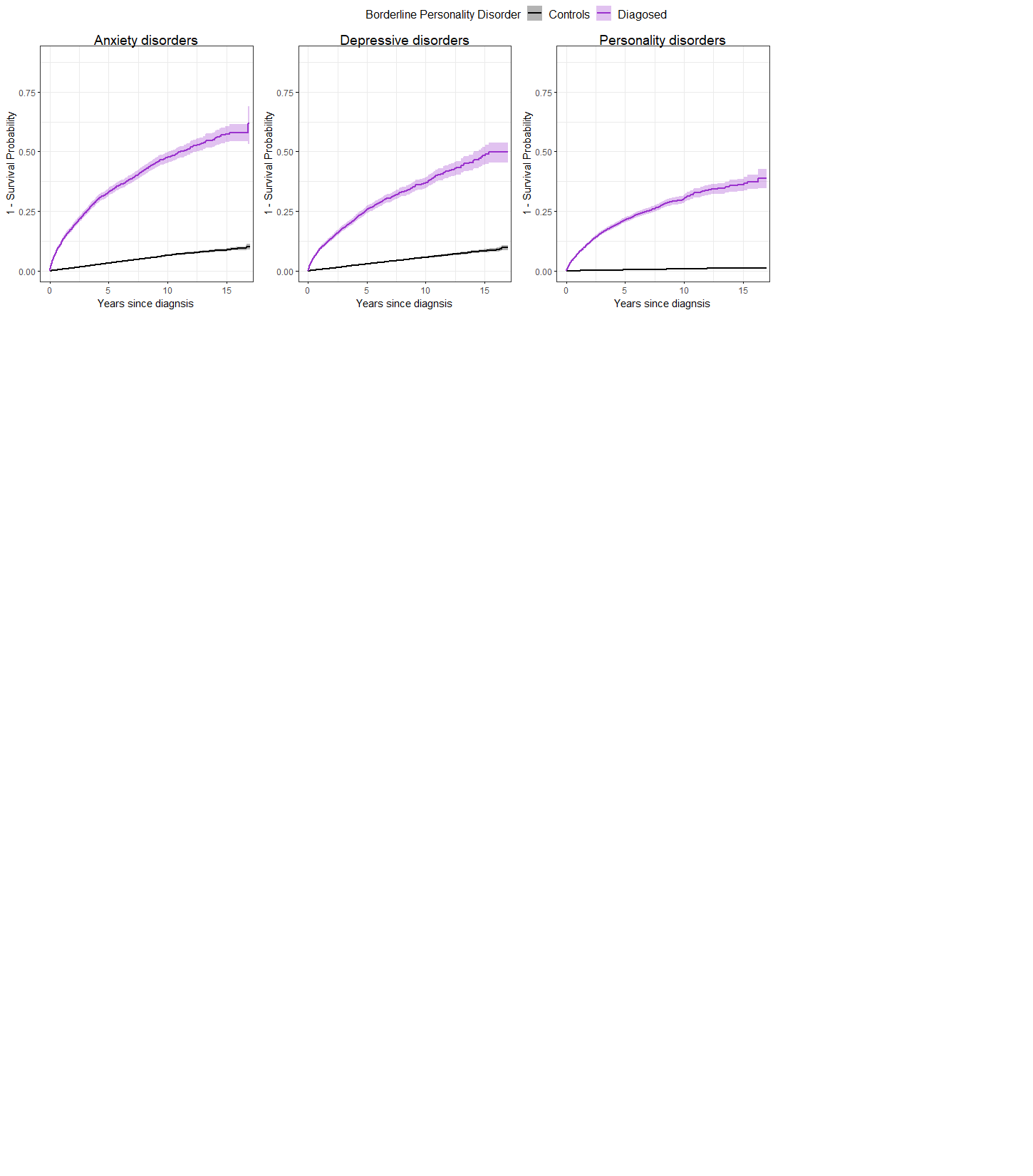


**Supplementary Figure 3. Cumulative incidence of psychiatric disorders with the second highest values during 17 years following a BPD diagnosis in the total sample, estimates and 95% confidence intervals shaded ^1^**


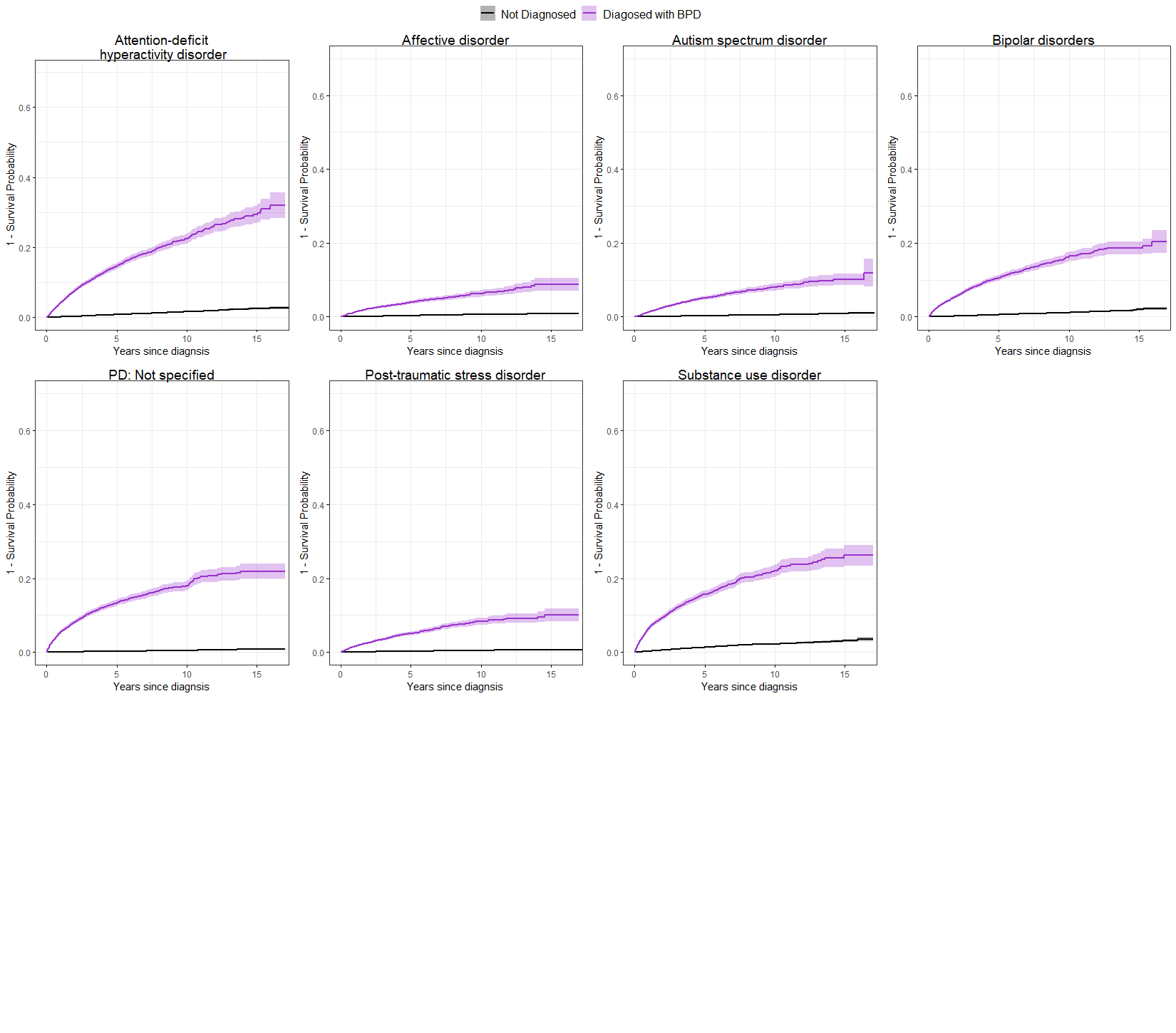


^1^ Personality Disorder (PD)

**Supplementary Figure 4. Cumulative incidence of psychiatric disorders with the lowest values during 17 years following a BPD diagnosis in the total sample, estimates and 95% confidence intervals (shaded) ^1^**


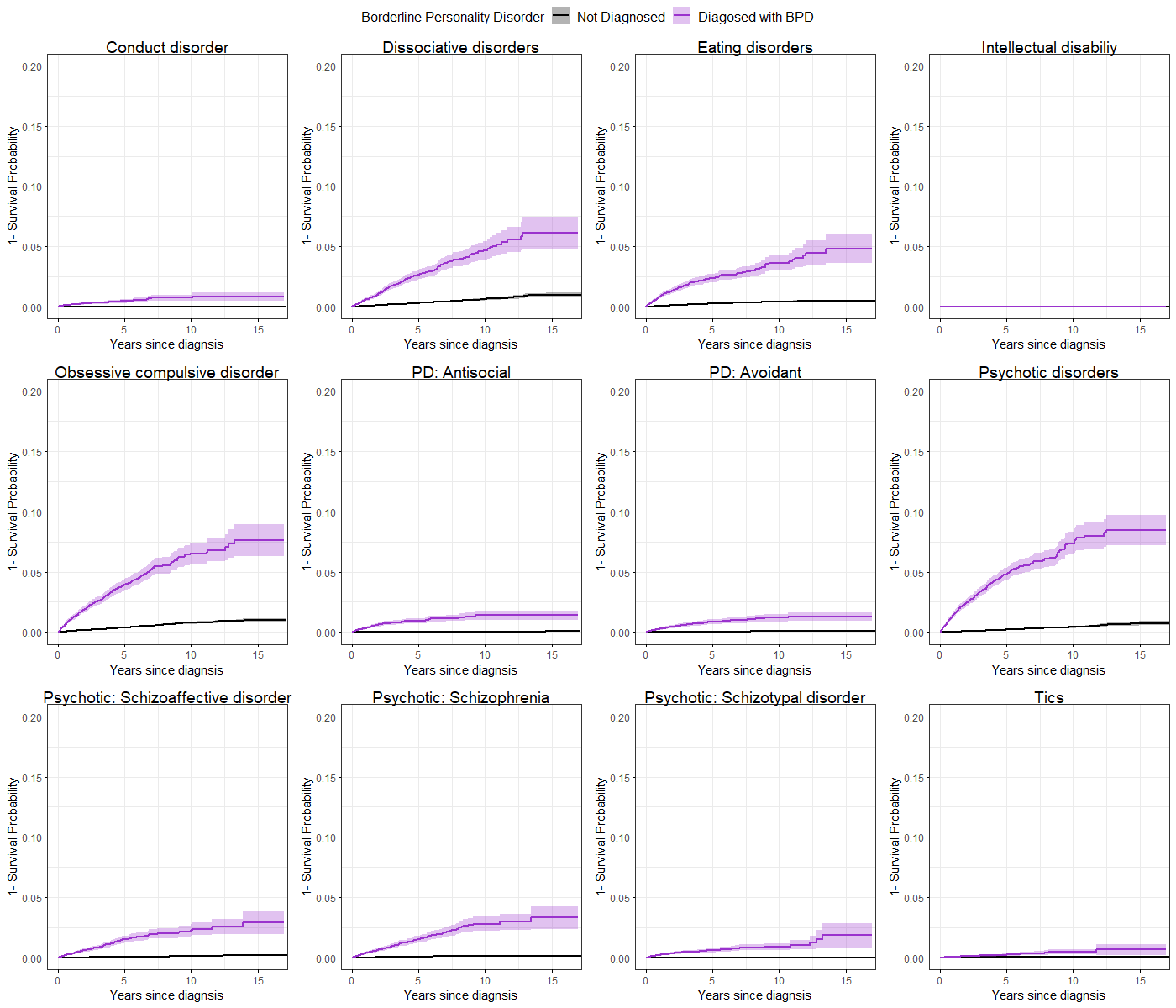


^1^ Personality Disorder (PD)

**Supplementary Figure 5. Cumulative incidence of somatic illnesses during 17 years following a BPD diagnosis in the total sample, estimates and 95% confidence intervals (shaded)^1^**


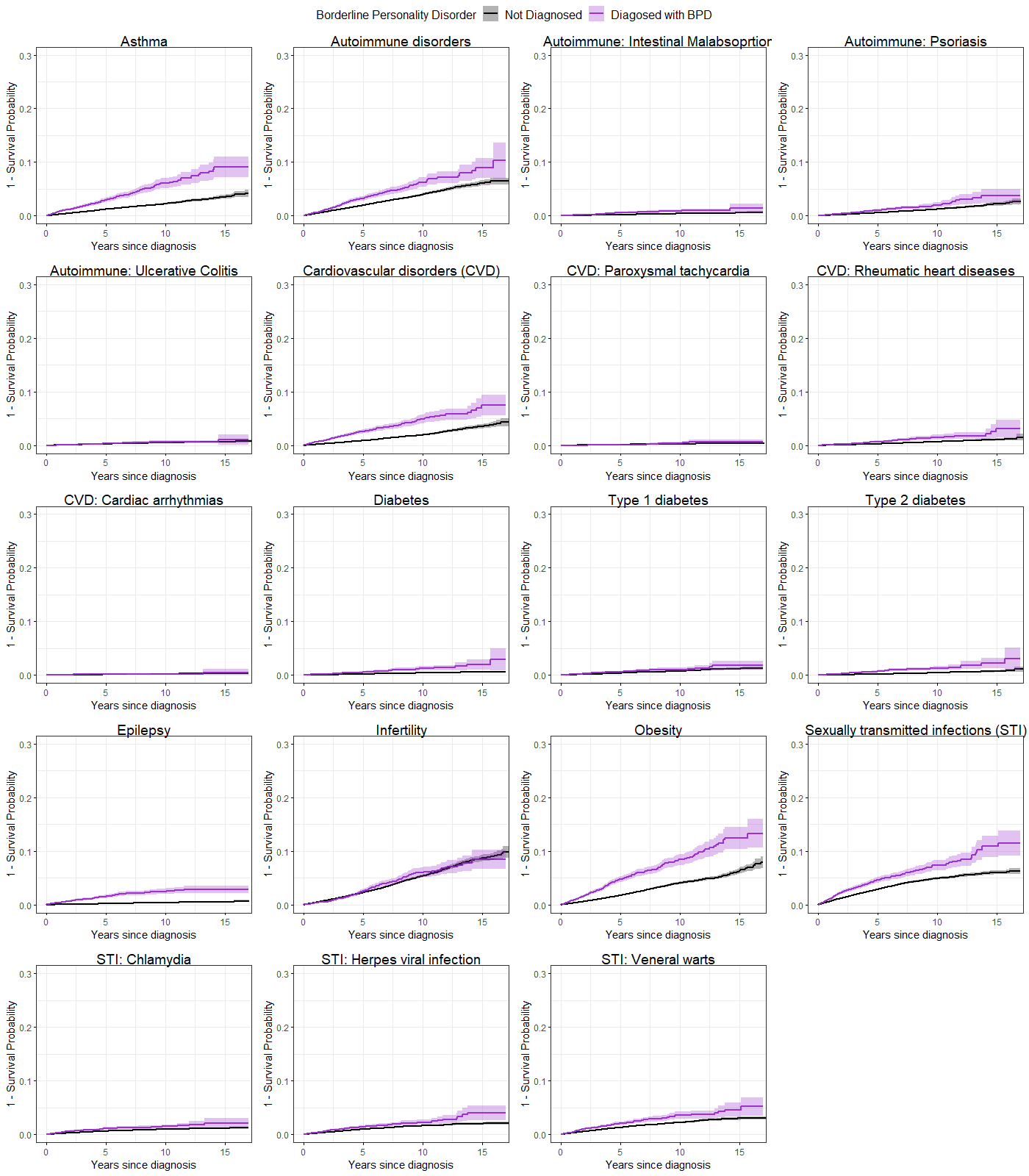


^1^ Abbreviations: Cardiovascular disorders (CVD), Sexually transmitted infection (STI),

**Supplementary Figure 6. Cumulative incidence of traumas and adverse behaviors with the highest values during 17 years following a BPD diagnosis in the total sample, estimates and 95% confidence intervals (shaded)**


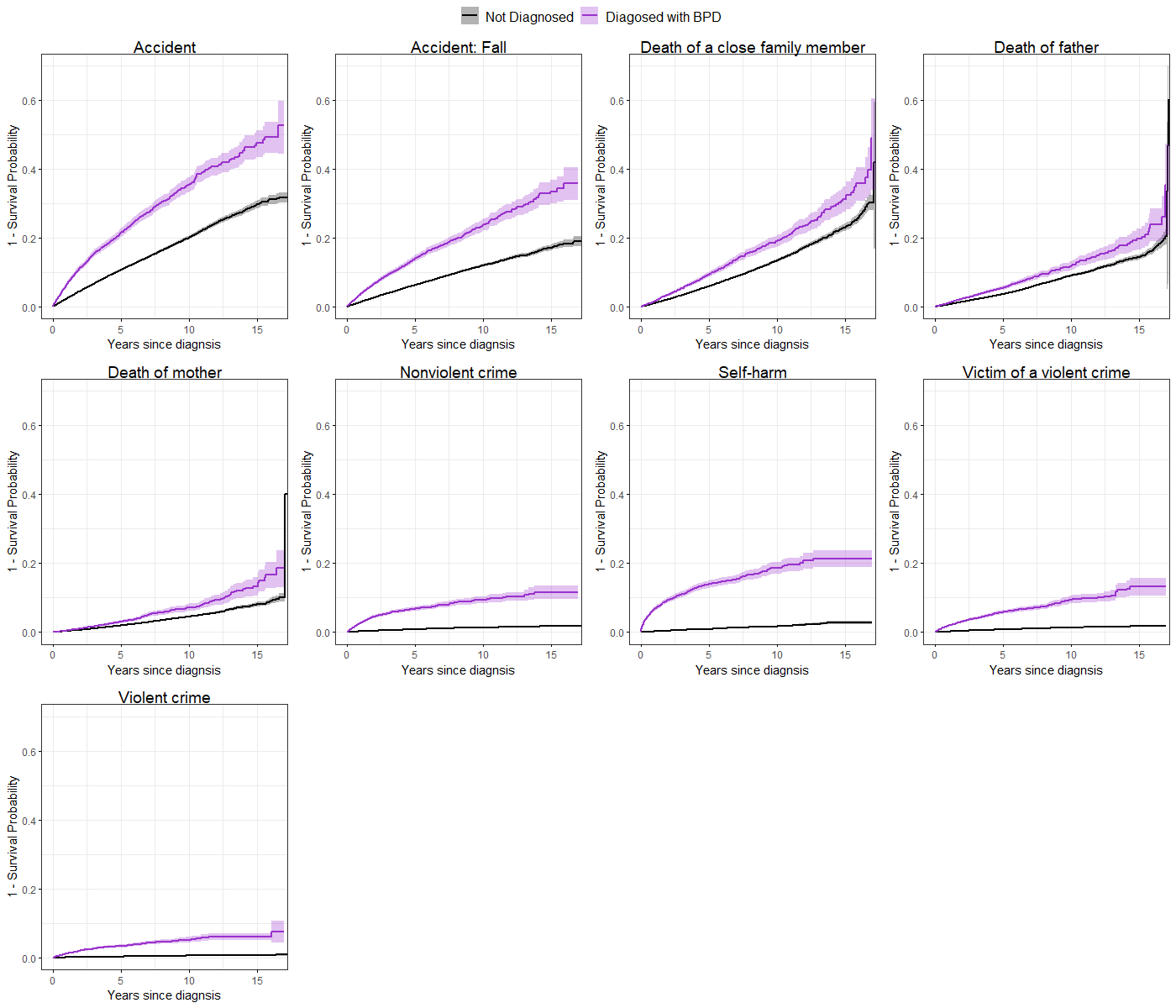


**Supplementary Figure 7. Cumulative incidence of traumas and adverse behaviors with the lowest values during 17 years following a BPD diagnosis in the total sample, estimates and 95% confidence intervals (shaded) ^1^**


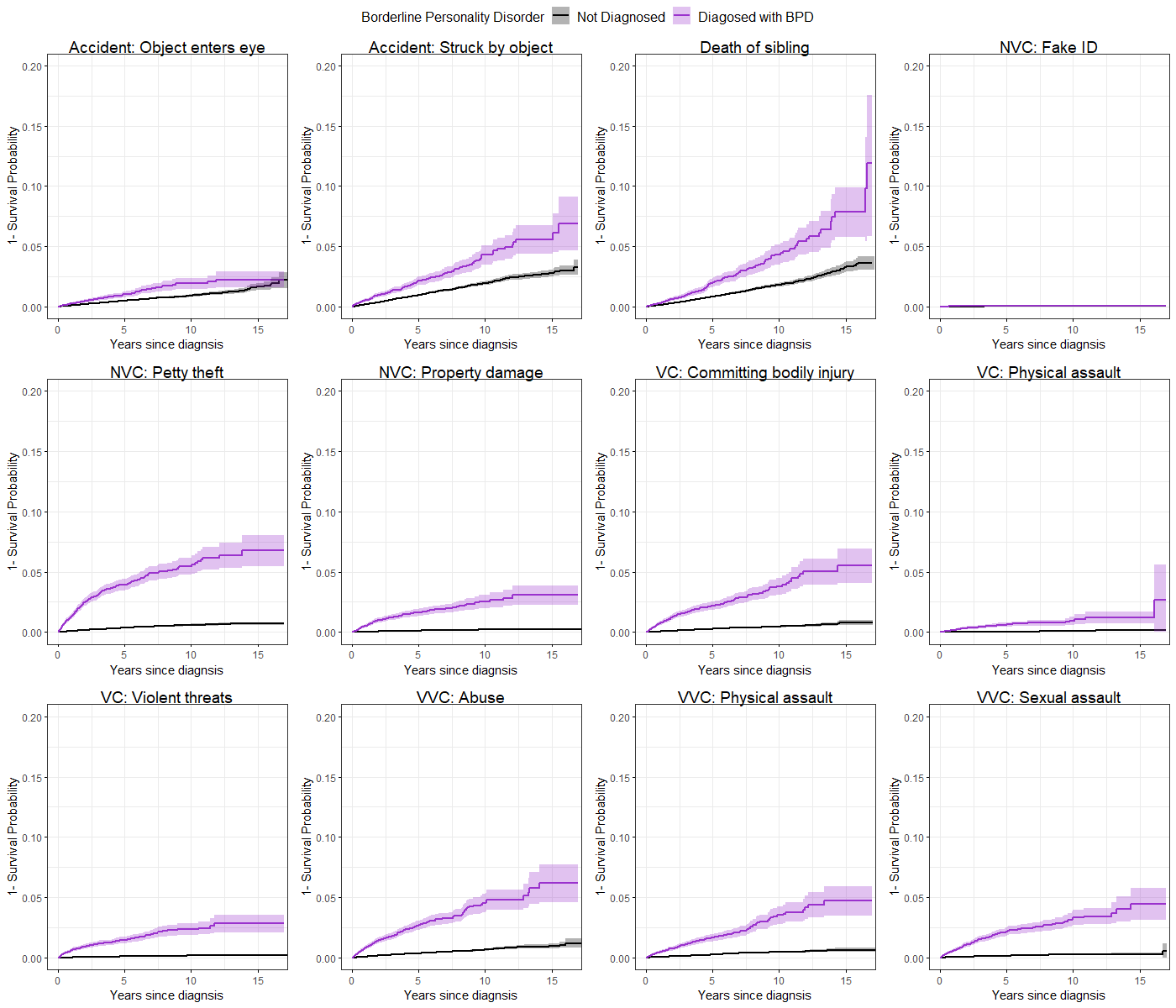


^a^ Abbreviations: Victim of a violent crime requiring medical attention (VVC), Violent crime conviction (VC), Nonviolent crime conviction (NVC)

**Supplementary Figure 8. Associations for psychiatric disorders and somatic disorder subcategories and Borderline Personality Disorder Diagnosis separated by sex, hazard ratio (95% confidence interval) ^1^**


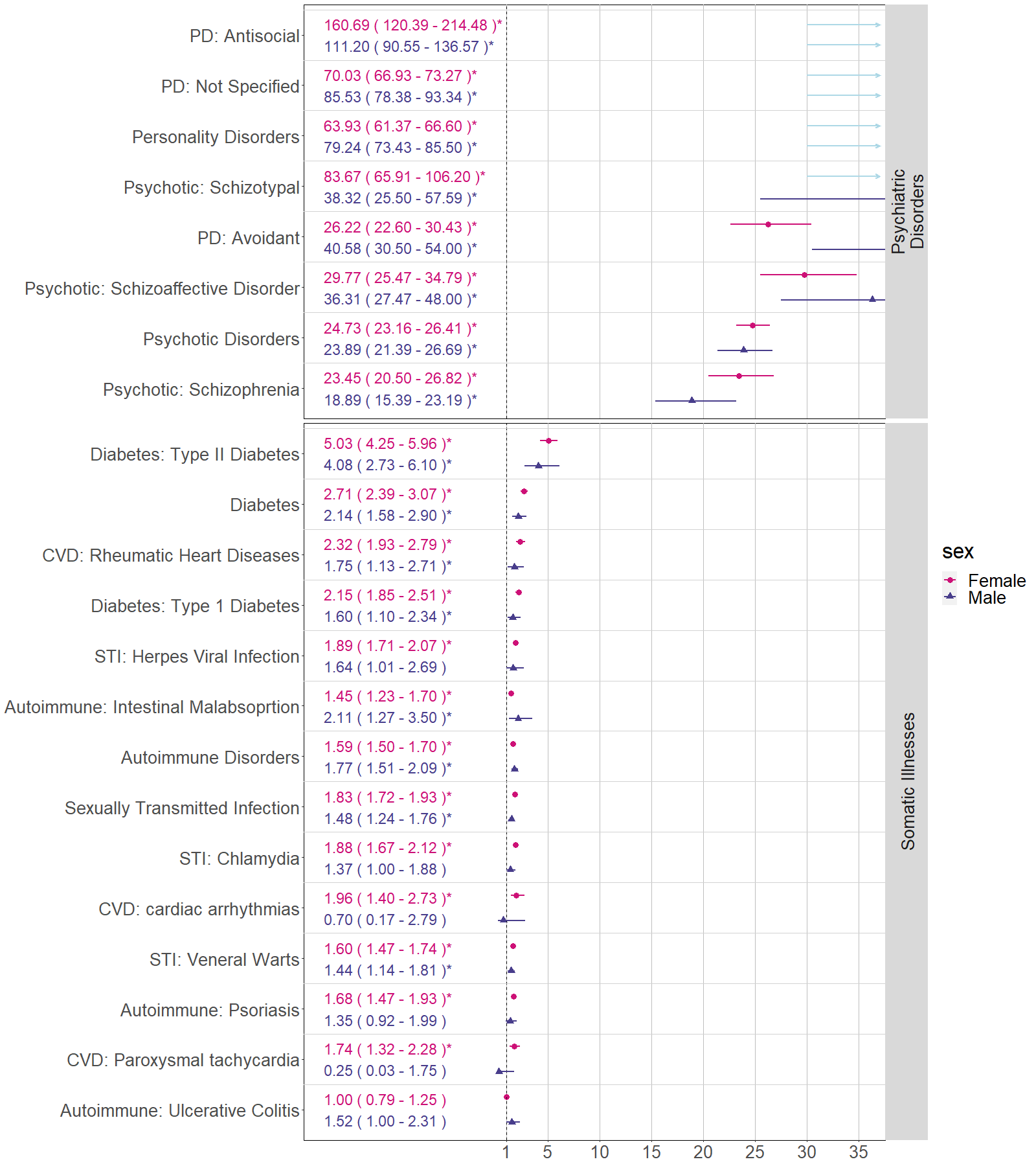


*Statistically significant after correcting for multiple testing

^1^ Abbreviations: Personality Disorder (PD)

**Supplementary Figure 9. Associations for trauma and behavior subcategories and Borderline Personality Disorder Diagnosis separated by sex, hazard ratio (95% confidence interval) ^1^**


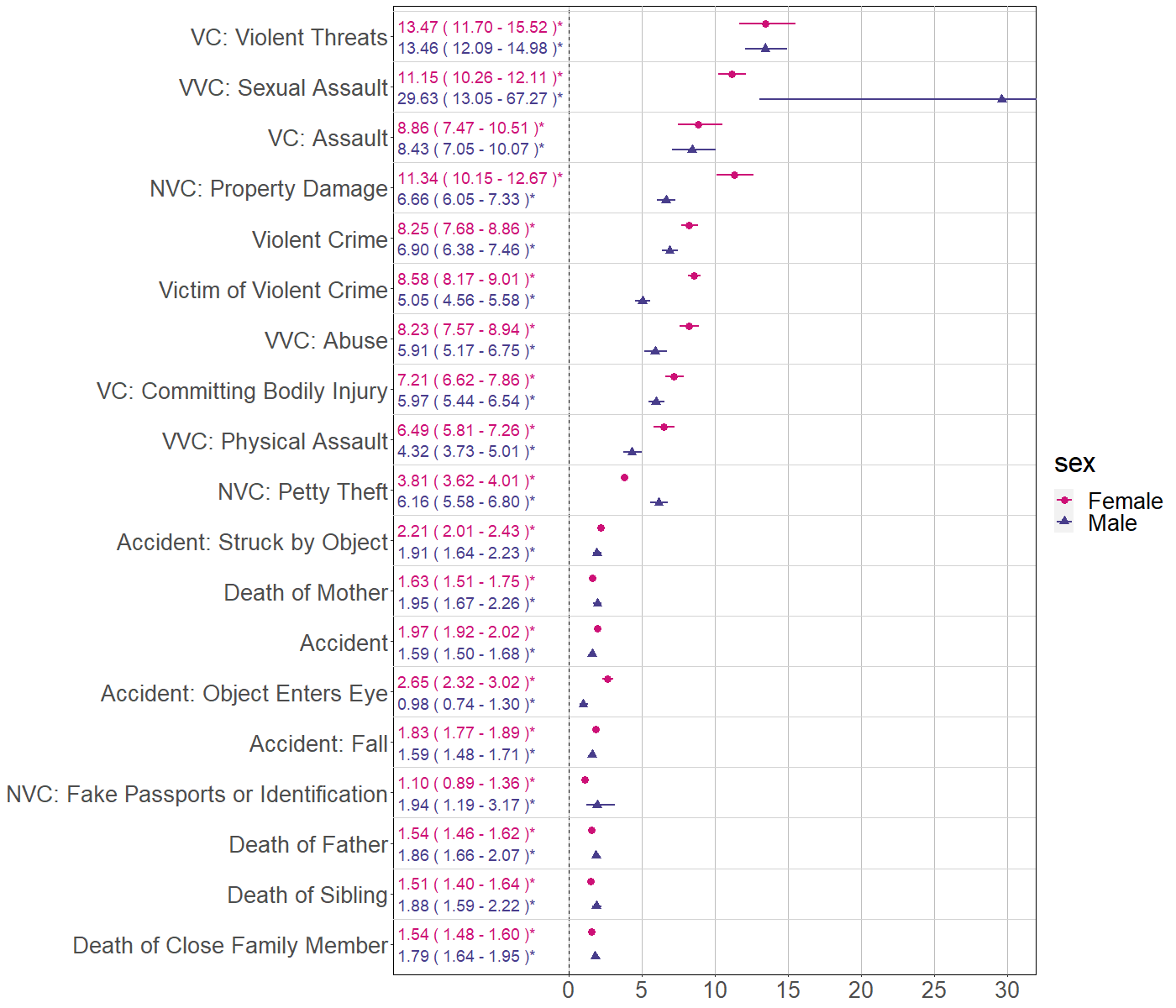


*Statistically significant after correcting for multiple testing

^1^ Abbreviations: Victim of a violent crime requiring medical attention (VVC), Violent crime conviction (VC), Nonviolent crime conviction (NVC)

**Supplementary Figure 10. Trauma and adverse behaviors as a risk factor and outcome for a Borderline Personality Disorder diagnosis, hazard ratio (95% confidence interval)^1^**


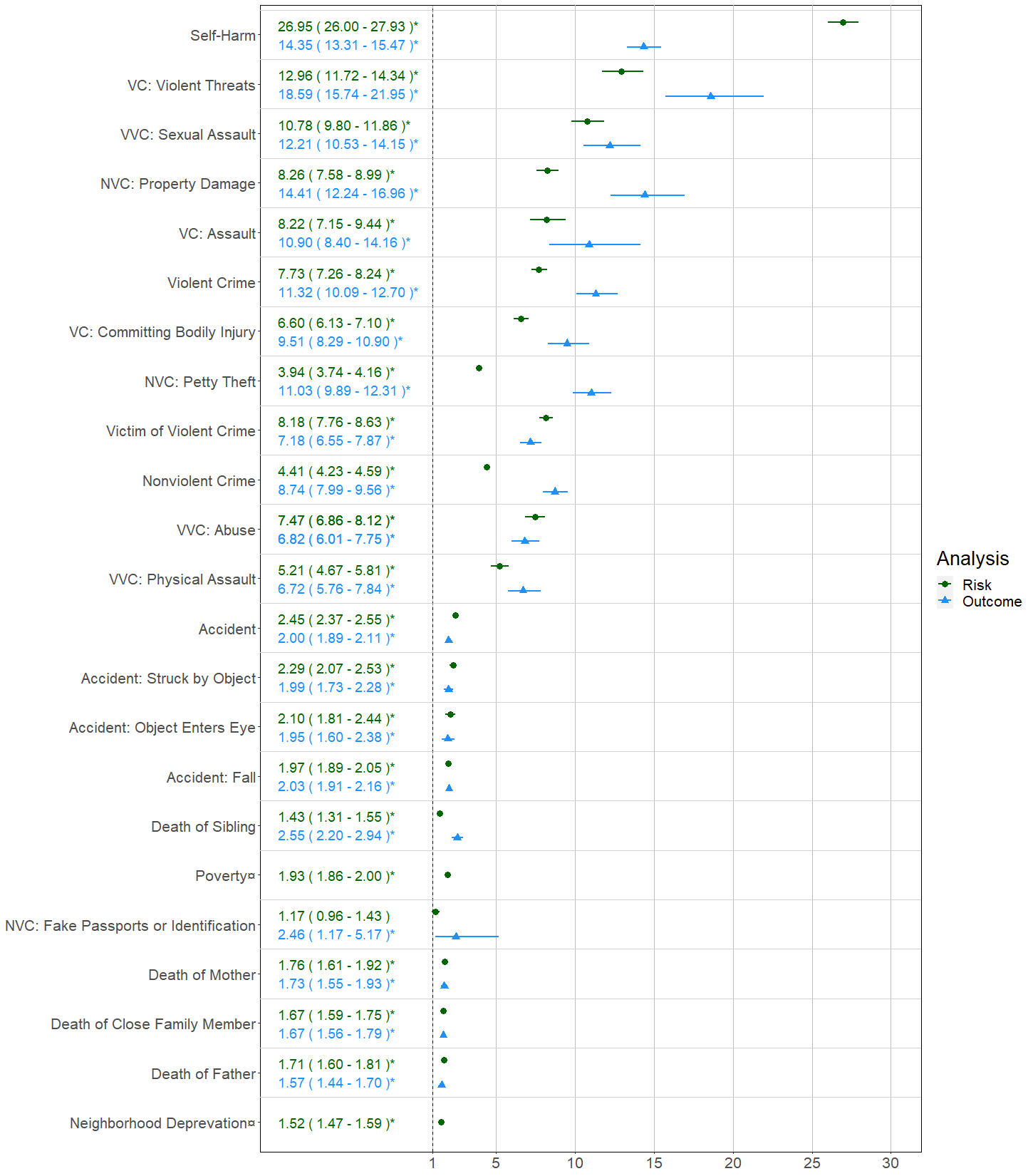


^a^ Risk indicates that the event occurred before a BPD diagnosis, outcome indicates that the event occurred after a BPD diagnosis

*Statistically significant after correcting for multiple testing

¤These variables were only measured in childhood

^1^ Abbreviations: Victim of a violent crime requiring medical attention (VVC), Violent crime conviction (VC), Nonviolent crime conviction (NVC)

**Supplementary Table 8. Trauma and adverse behaviors as a risk factor or outcome for a Borderline Personality Disorder diagnosis, hazard ratio (95% confidence interval)**

|  | **Males**  **Risk factor prior to BPD diagnosis** | | **Females**  **Risk factor prior to BPD diagnosis** | | **Males**  **Outcomes following BPD diagnosis** | | **Females**  **Outcomes following BPD diagnosis** | |
| --- | --- | --- | --- | --- | --- | --- | --- | --- |
| Accident | 2.11 | (1.92-2.32) ^*^ | 2.52 | (2.42-2.62) ^*^ | 1.43 | (1.24-1.65) ^*^ | 2.13 | (2.00-2.26) ^*^ |
| Accident: Fall | 1.74 | (1.57-1.93) ^*^ | 2.02 | (1.93-2.11) ^*^ | 1.79 | (1.55-2.07) ^*^ | 2.07 | (1.93-2.22) ^*^ |
| Accident: Object enters eye | 0.85 | (0.57-1.27) | 2.83 | (2.40-3.33) ^*^ | 1.14 | (0.76-1.72) | 2.39 | (1.90-2.99) ^*^ |
| Accident: Struck by object | 2.25 | (1.86-2.72) ^*^ | 2.36 | (2.10-2.65) ^*^ | 1.55 | (1.18-2.04) ^*^ | 2.15 | (1.83-2.51) ^*^ |
| Death of a close family member | 1.89 | (1.68-2.12) ^*^ | 1.63 | (1.54-1.72) ^*^ | 1.96 | (1.67-2.29) ^*^ | 1.61 | (1.49-1.74) ^*^ |
| Death of father | 2.04 | (1.76-2.35) ^*^ | 1.65 | (1.54-1.76) ^*^ | 1.52 | (1.38-1.66) ^*^ | 1.82 | (1.50-2.21) ^*^ |
| Death of mother | 2.06 | (1.69-2.51) ^*^ | 1.70 | (1.55-1.87) ^*^ | 1.98 | (1.55-2.54) ^*^ | 1.68 | (1.49-1.90) ^*^ |
| Death of sibling | 1.54 | (1.25-1.9) ^*^ | 1.41 | (1.28-1.54) ^*^ | 3.80 | (2.83-5.11) ^*^ | 2.31 | (1.95-2.72) ^*^ |
| Neighborhood deprivation during childhood^1^ | 1.82 | (1.64-2.01) ^*^ | 1.48 | (1.42-1.54) ^*^ | NA |  | NA |  |
| Nonviolent crime (NV) | 5.89 | (5.36-6.48) ^*^ | 4.08 | (3.89-4.28) ^*^ | 7.59 | (6.20-9.29) ^*^ | 8.32 | (7.53-9.19) ^*^ |
| NV: Fake passports or identification | 1.97 | (1.19-3.28) | 1.09 | (0.87-1.35) | 2.81 | (0.39-19.93) | 2.34 | (1.05-5.21) ^*^ |
| NV: Petty theft | 6.13 | (5.42-6.93) ^*^ | 9.64 | (8.52-10.9) ^*^ | 15.49 | (12.41-19.33) ^*^ | 9.58 | (8.45-10.87) ^*^ |
| NV: Property damage | 7.22 | (6.43-8.11) ^*^ | 3.61 | (3.41-3.83) ^*^ | 9.85 | (7.64-12.7) ^*^ | 20.35 | (16.37-25.31) ^*^ |
| Poverty during childhood^1^ | 2.31 | (2.1-2.55) ^*^ | 1.87 | (1.79-1.95) ^*^ | N* |  | N* |  |
| Self-harm | 22.46 | (20.40-24.73) ^*^ | 27.82 | (26.76-28.92) ^*^ | 13.54 | (11.41-16.07) ^*^ | 14.73 | (13.54-16.01) ^*^ |
| Victim of violent crime (VVC) | 66.04 | (5.31-6.86) ^*^ | 88.94 | (8.44-9.47) ^*^ | 44.16 | (3.37-5.15) ^*^ | 99.29 | (8.4-10.29) ^*^ |
| VVC: Abuse | 77.05 | (6.00-8.27) ^*^ | 77.86 | (7.12-8.68) ^*^ | 44.41 | (3.35-5.81) ^*^ | 88.28 | (7.17-9.56) ^*^ |
| VVC: Physical Assault | 44.07 | (3.37-4.91) ^*^ | 66.22 | (5.44-7.11) ^*^ | 55.65 | (4.37-7.32) ^*^ | 77.29 | (6.01-8.83) ^*^ |
| VVC: Sexual Assault | 225.08 | (9.4-66.92) ^*^ | 110.57 | (9.60-11.63) ^*^ | NNA |  | 112.07 | (10.40 – 14.00) ^*^ |
| Violent crime (VC) | 88.01 | (7.23-8.88) ^*^ | 77.56 | (6.97-8.20) ^*^ | 99.35 | (7.73-11.32) ^*^ | 111.75 | (10.16 -13.58) ^*^ |
| VC: Assault | 88.24 | (6.68-10.16) ^*^ | 88.18 | (6.80-9.85) ^*^ | 110.45 | (7.20-15.16) ^*^ | 110.47 | (7.25-15.12) ^*^ |
| VC: Committing bodily injury | 66.56 | (5.85-7.36) ^*^ | 66.61 | (6.00-7.28) ^*^ | 77.40 | (5.92-9.26) ^*^ | 110.38 | (8.72-12.36) ^*^ |
| VC: Threats of violence | 14.36 | (12.59-16.38) ^*^ | 11.41 | (9.73-13.37) ^*^ | 15.99 | (12.79-19.99) ^*^ | 20.58 | (15.96-26.54) ^*^ |

^a^ Significant after correcting for multiple testing

**Supplementary Figure 11. Cumulative incidence of psychiatric disorders by sex for 5 years after Borderline Personality Disorder diagnosis, estimate and (95% confidence interval) ^1^**


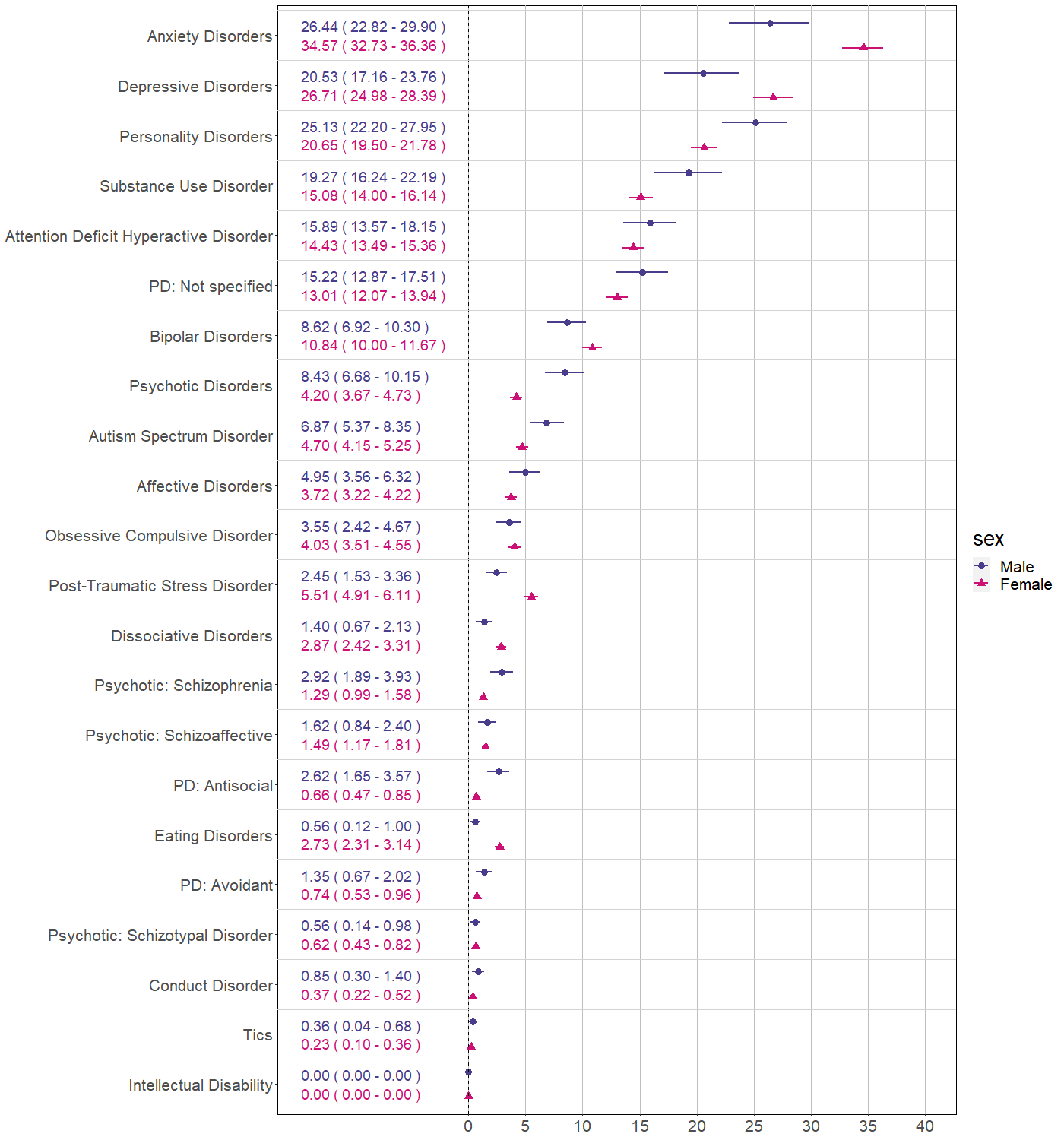


^1^ Abbreviation: Personality Disorder (PD)

**Supplementary Figure 12. Cumulative incidence of somatic illnesses by sex for 5 years after Borderline Personality Disorder diagnosis, estimate and (95% confidence interval) ^1^**


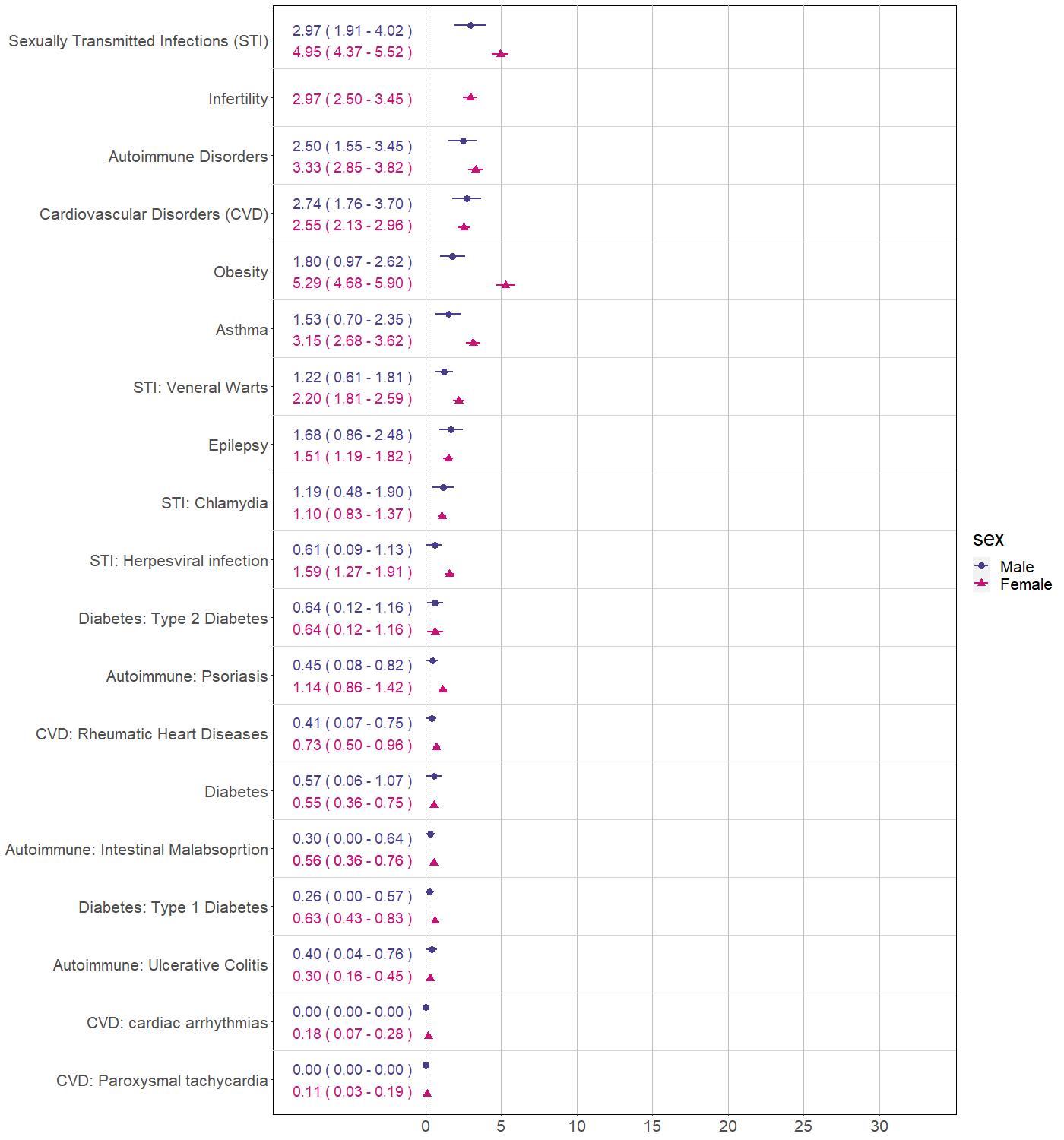


^1^ Abbreviations: Cardiovascular disorders (CVD), Sexually transmitted infection (STI)

**Supplementary Figure 13 Cumulative incidence of trauma and adverse behaviors by sex for 5 years after Borderline Personality Disorder diagnosis, estimate and (95% confidence interval)^1^**


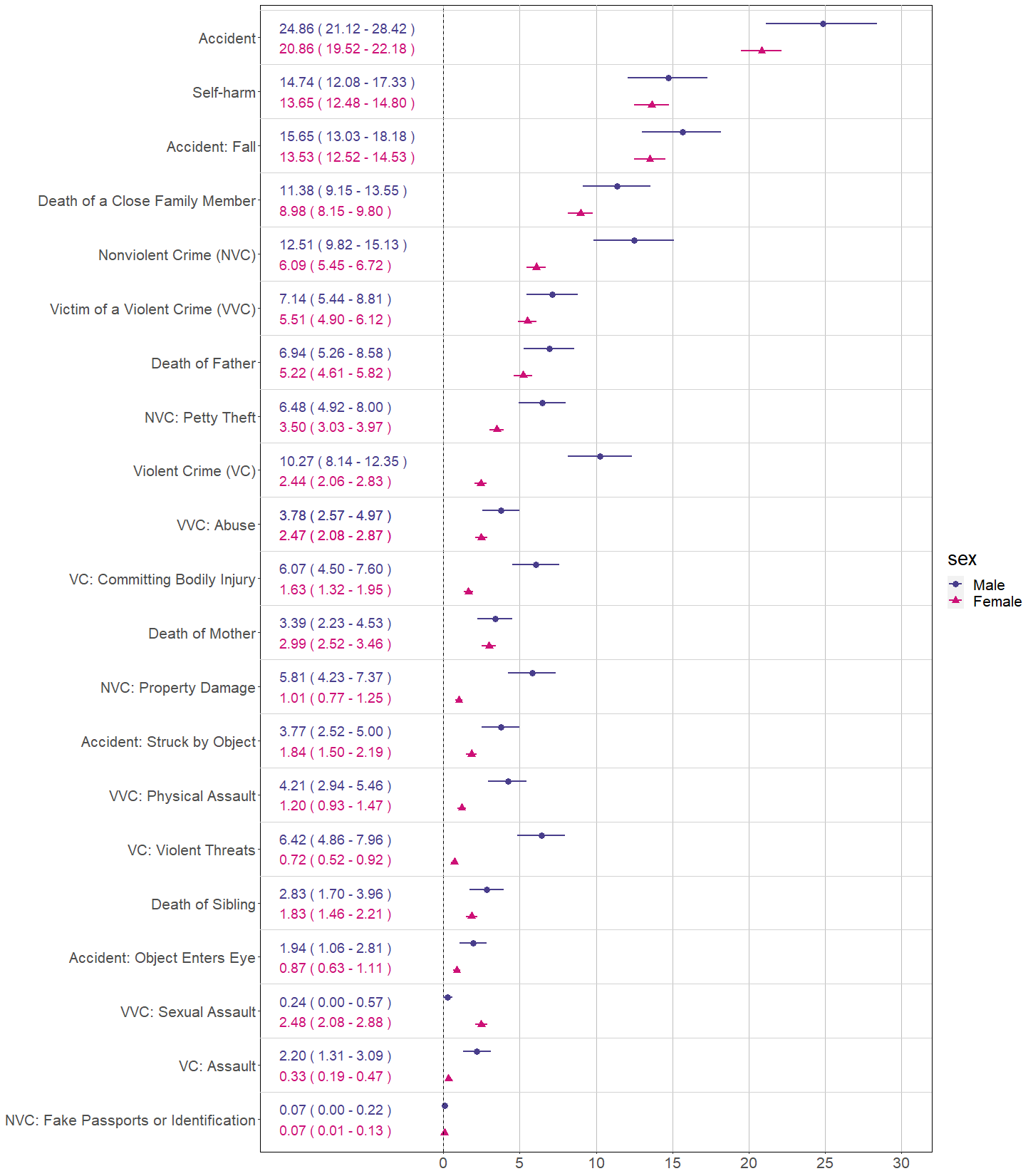


^1^ Abbreviations: Victim of a violent crime requiring medical attention (VVC), Violent crime conviction (VC), Nonviolent crime conviction (NVC)

**Supplementary Figure 14. Cumulative incidence of psychiatric disorders with the highest values during 17 years following a BPD diagnosis in males, estimates and 95% confidence intervals (shaded) ^1^**


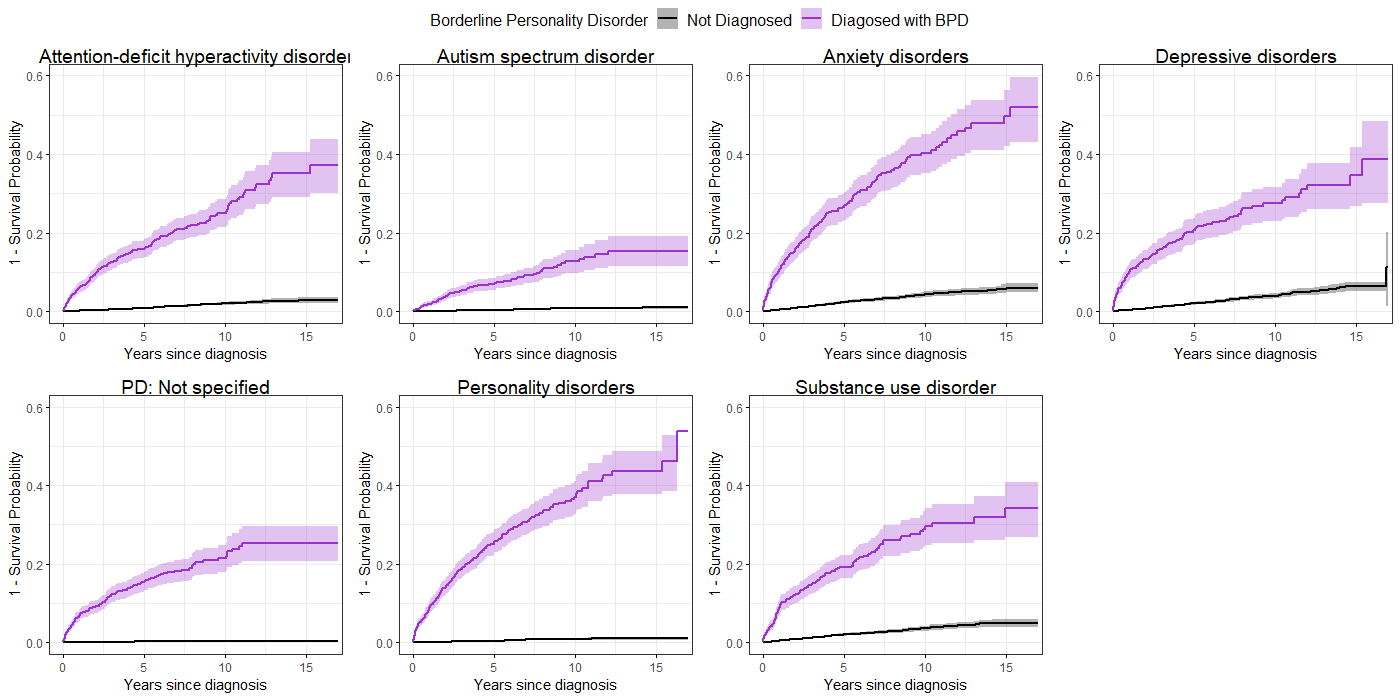


**^1^** Personality disorder (PD)

**Supplementary Figure 15. Cumulative incidence of psychiatric disorders with the lowest values during 17 years following a BPD diagnosis in males, estimates and 95% confidence intervals (shaded) ^1^**


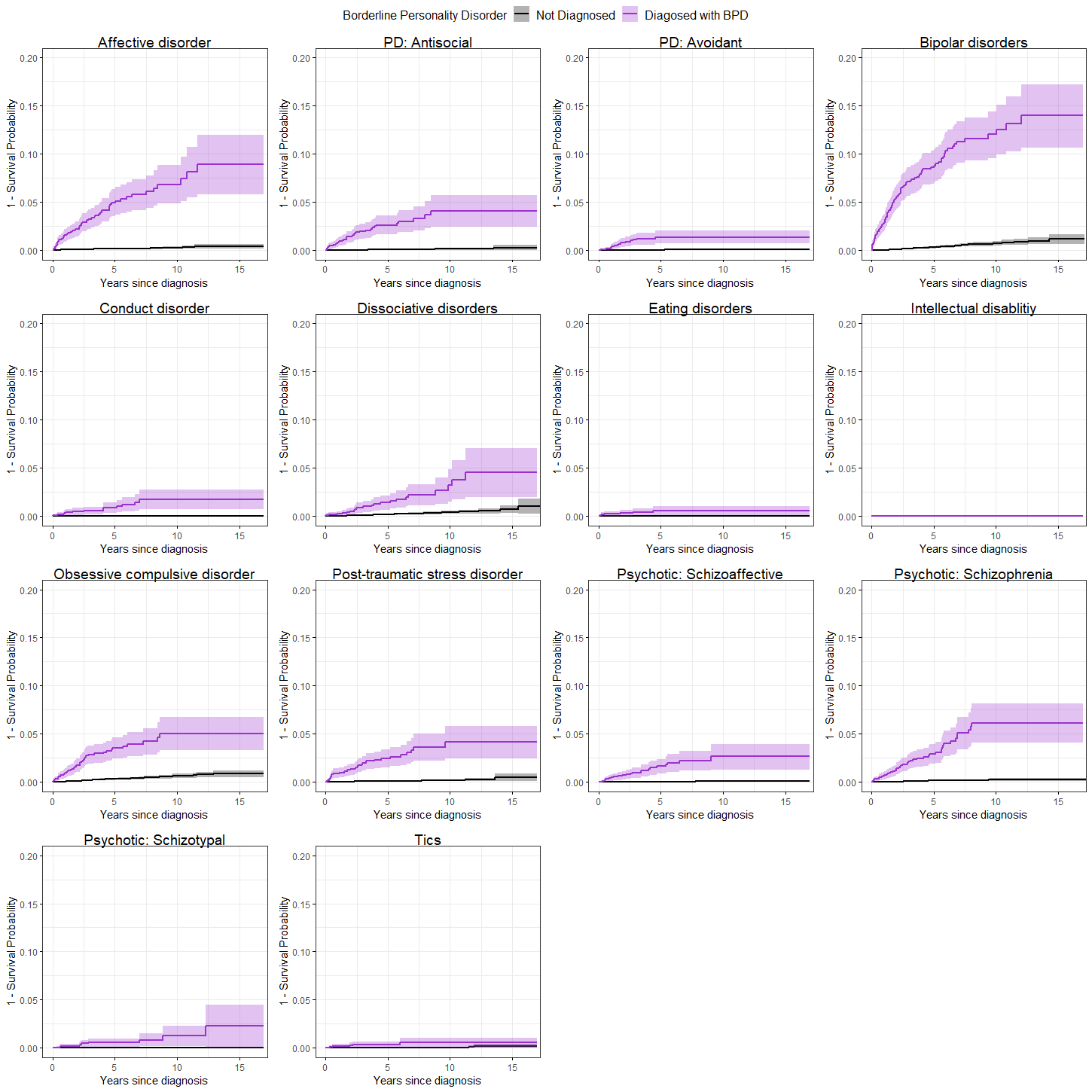


^1^ Personality disorder (PD)

**Supplementary Figure 16. Cumulative incidence of psychiatric disorders with the highest values during 17 years following a BPD diagnosis in females, estimates and 95% confidence intervals (shaded)^1^**


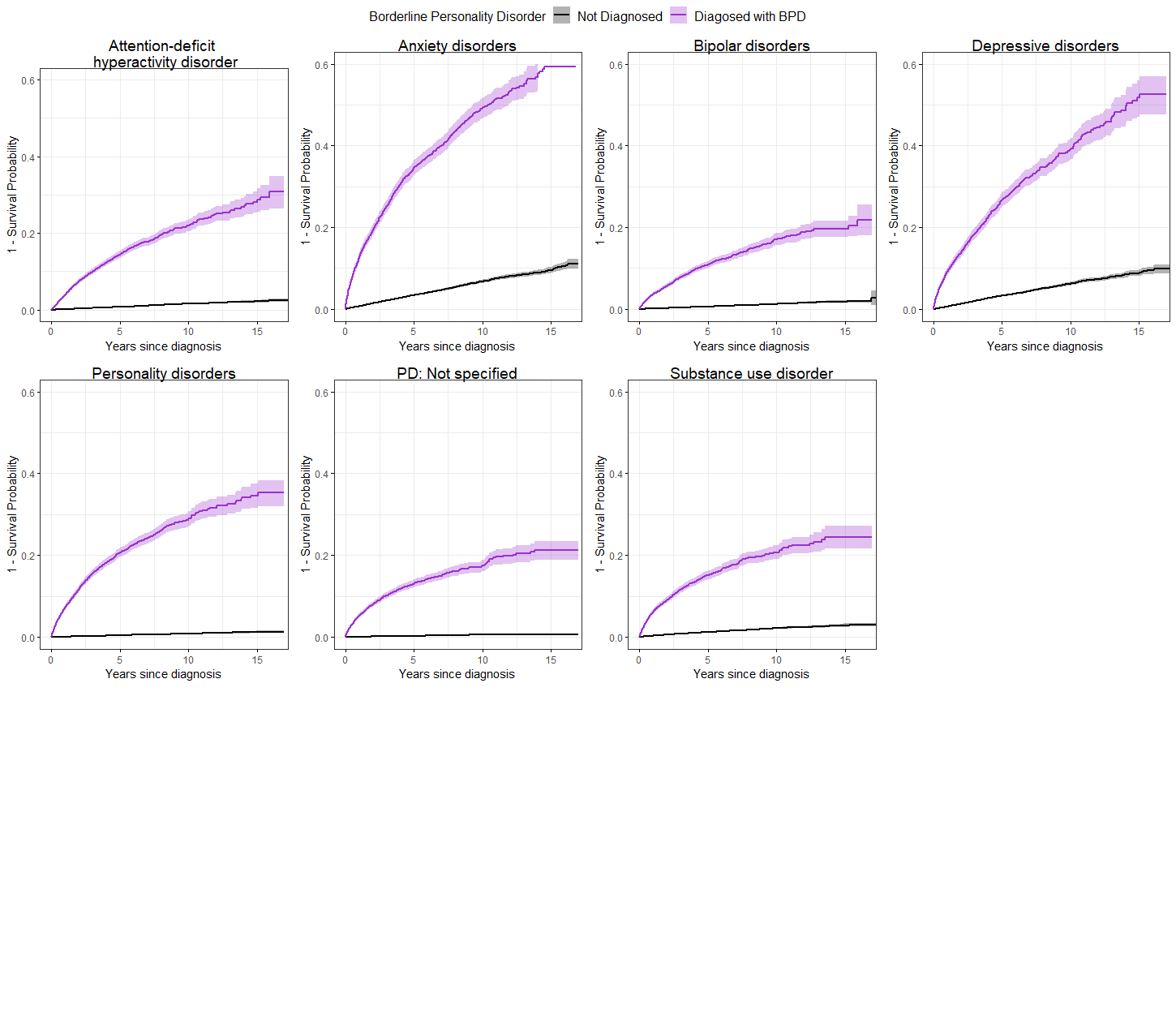


^1^ Personality disorders (PD)

**Supplementary Figure 17. Cumulative incidence of psychiatric disorders with the lowest values during 17 years following a BPD diagnosis in females, estimates and 95% confidence intervals (shaded) ^1^**


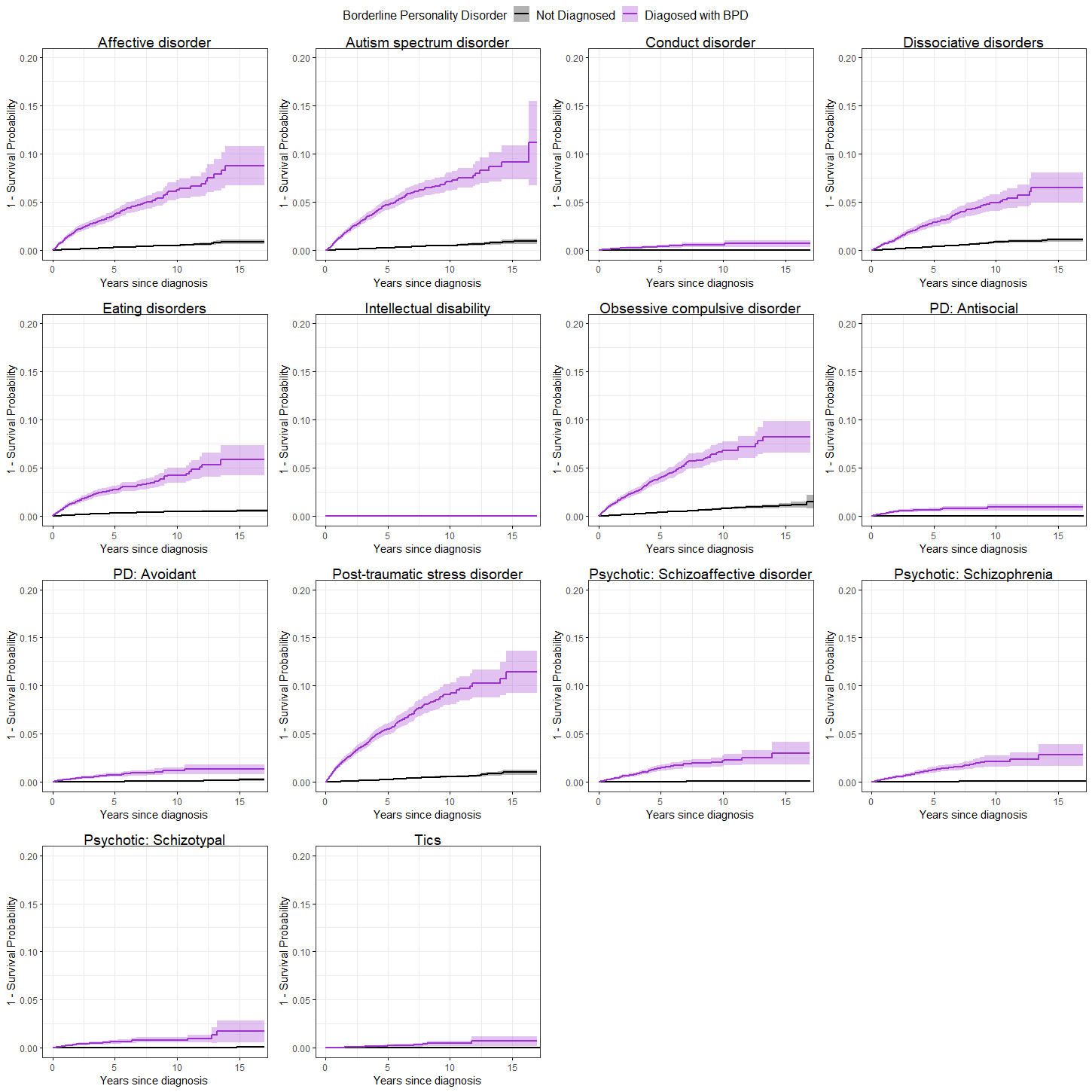


^1^ Personality Disorders (PD)**Supplementary Figure 18. Cumulative incidence of somatic illnesses during 17 years following a BPD diagnosis in males, estimates and 95% confidence intervals (shaded)**


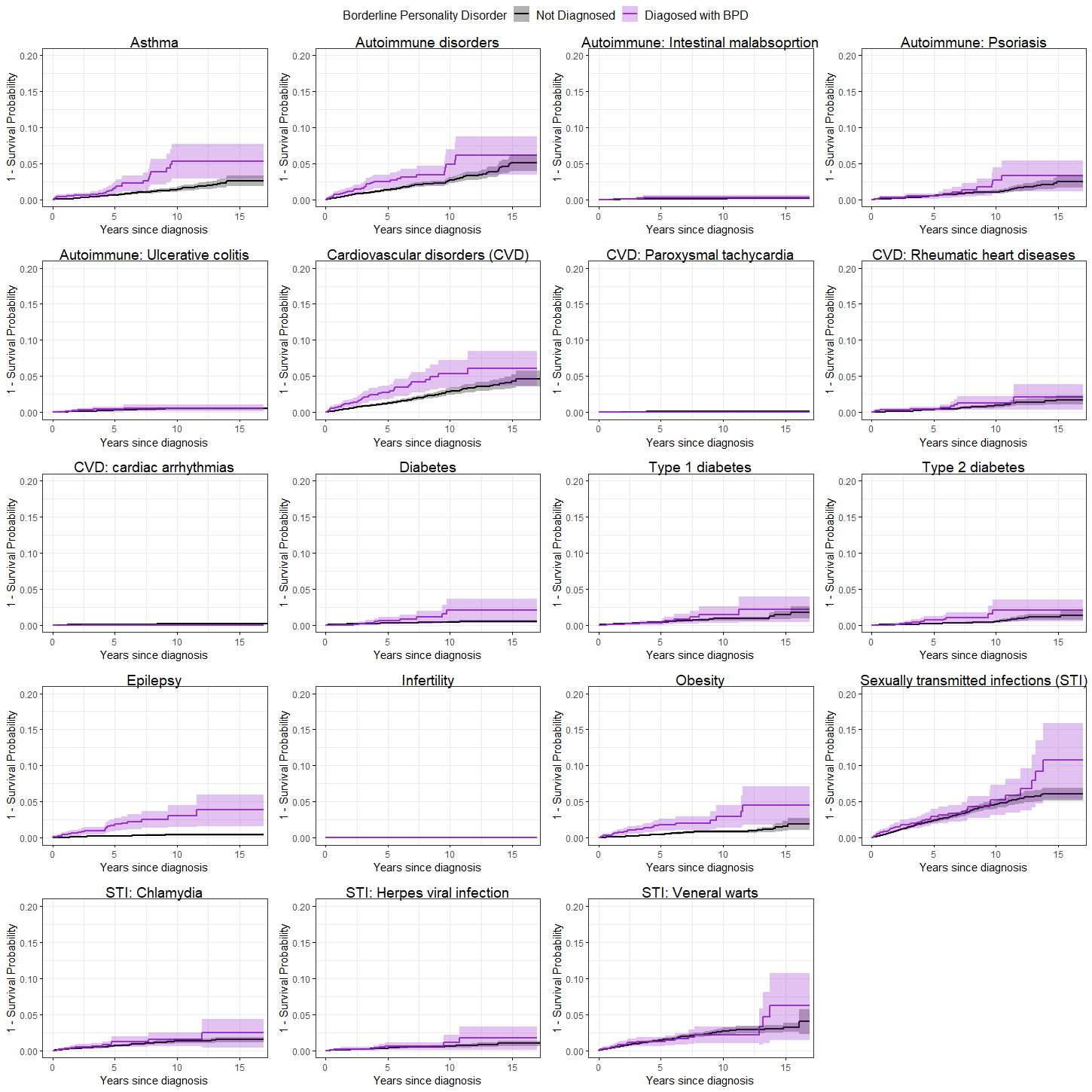


**Supplementary Figure 19. Cumulative incidence of somatic illnesses during 17 years following a BPD diagnosis in females, estimates and 95% confidence intervals (shaded)**


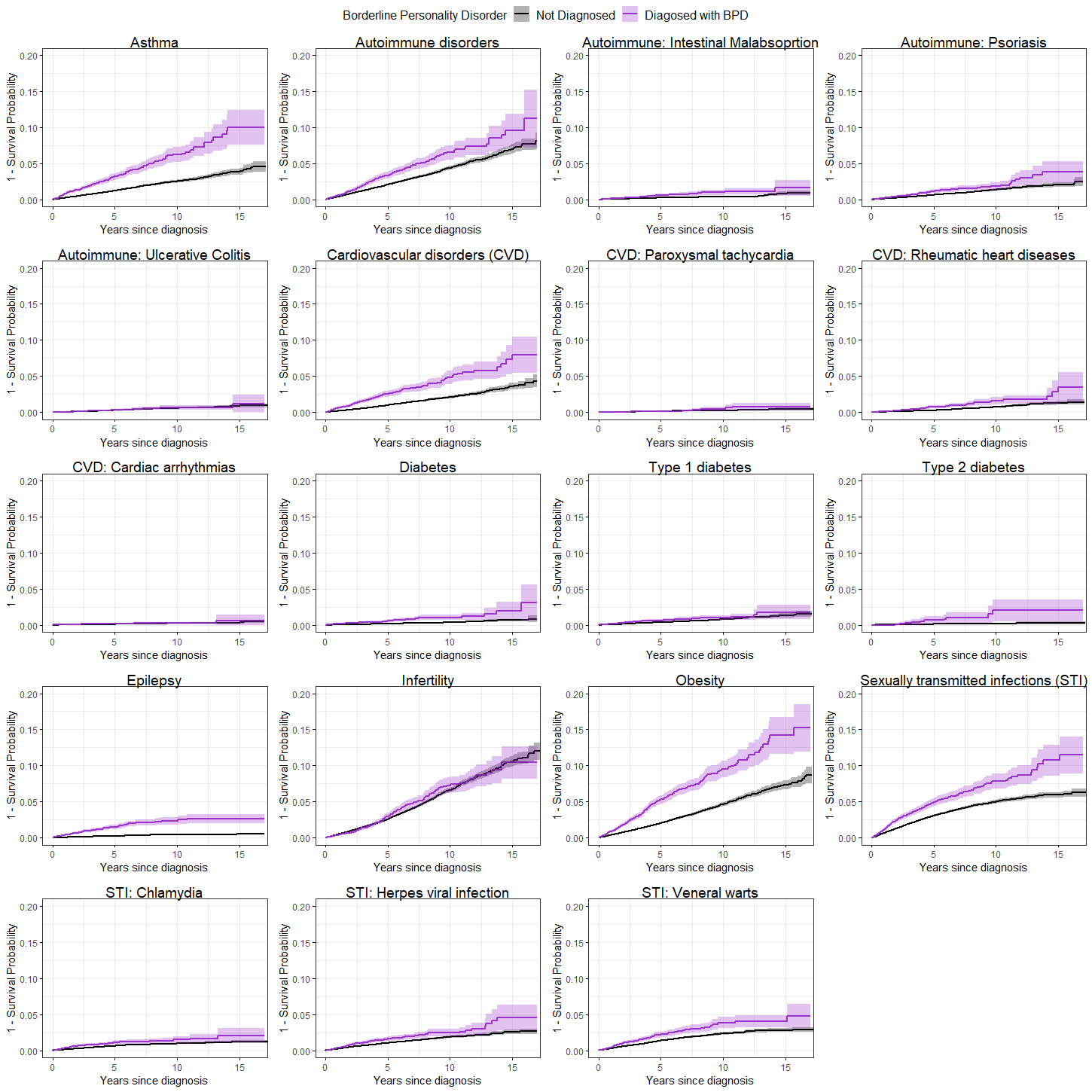


**Supplementary Figure 20. Cumulative incidence of traumas and adverse behaviors with the highest values during 17 years following a BPD diagnosis in males, estimates and 95% confidence intervals (shaded)**


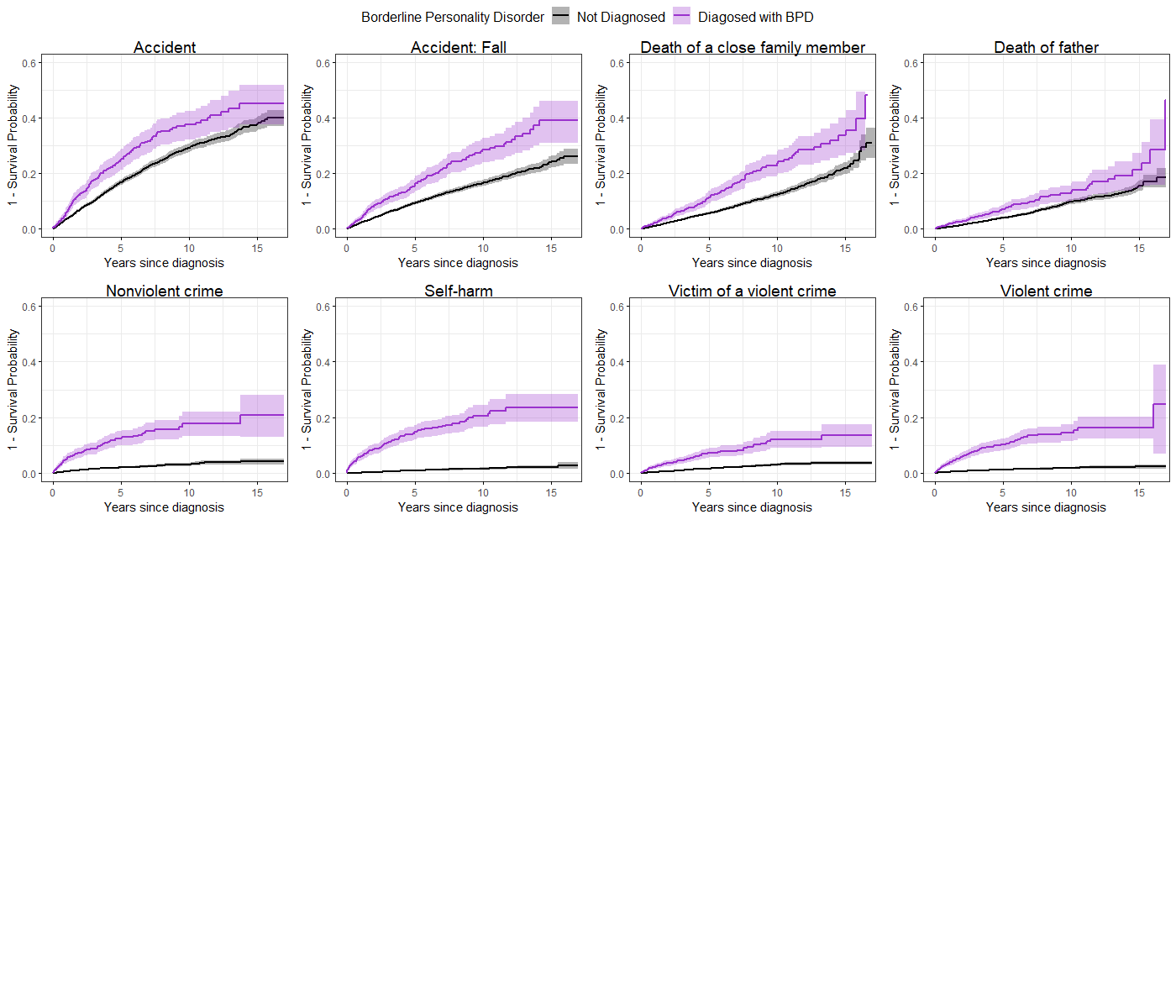


**Supplementary Figure 21. Cumulative incidence of traumas and adverse behaviors with the lowest values during 17 years following a BPD diagnosis in males, estimates and 95% confidence intervals (shaded) ^1^**


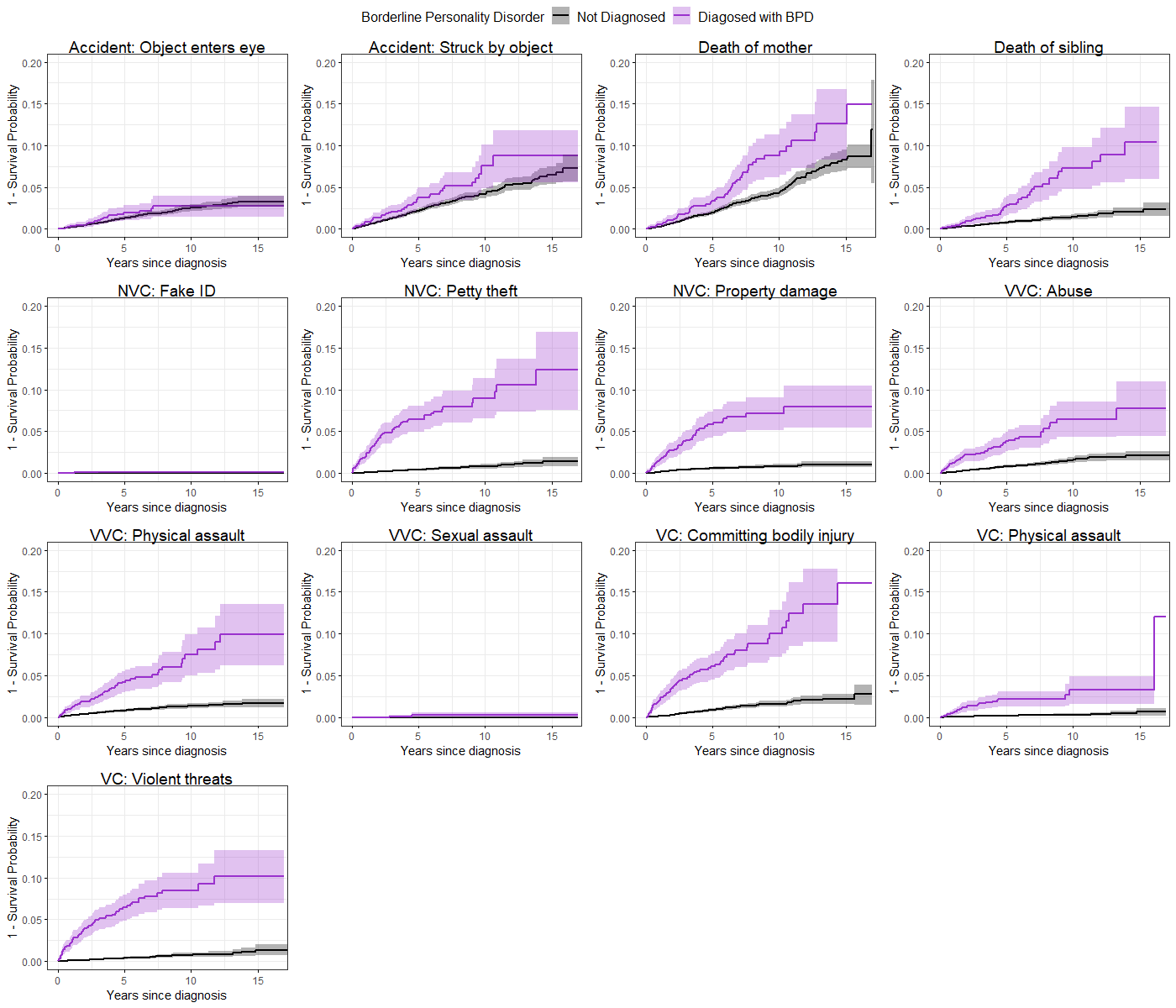


^1^ Abbreviations: Victim of a violent crime requiring medical attention (VVC), Violent crime conviction (VC), Nonviolent crime conviction (NVC)

**Supplementary Figure 22. Cumulative incidence of traumas and adverse behaviors with the highest values during 17 years following a BPD diagnosis in females, estimates and 95% confidence intervals (shaded)**


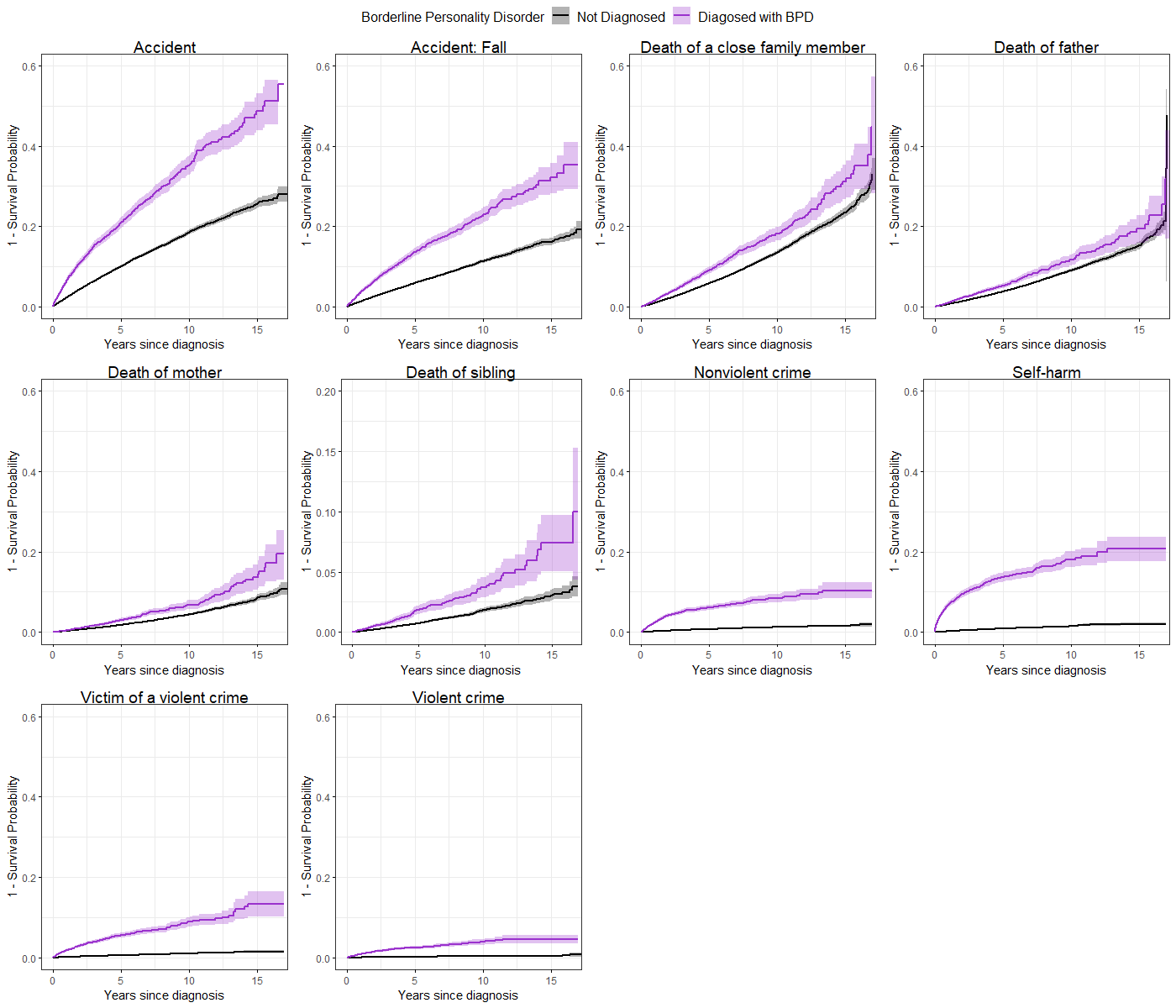


**Supplementary Figure 23. Cumulative incidence of traumas and adverse behaviors with the lowest values during 17 years following a BPD diagnosis in females, estimates and 95% confidence intervals (shaded) ^1^**


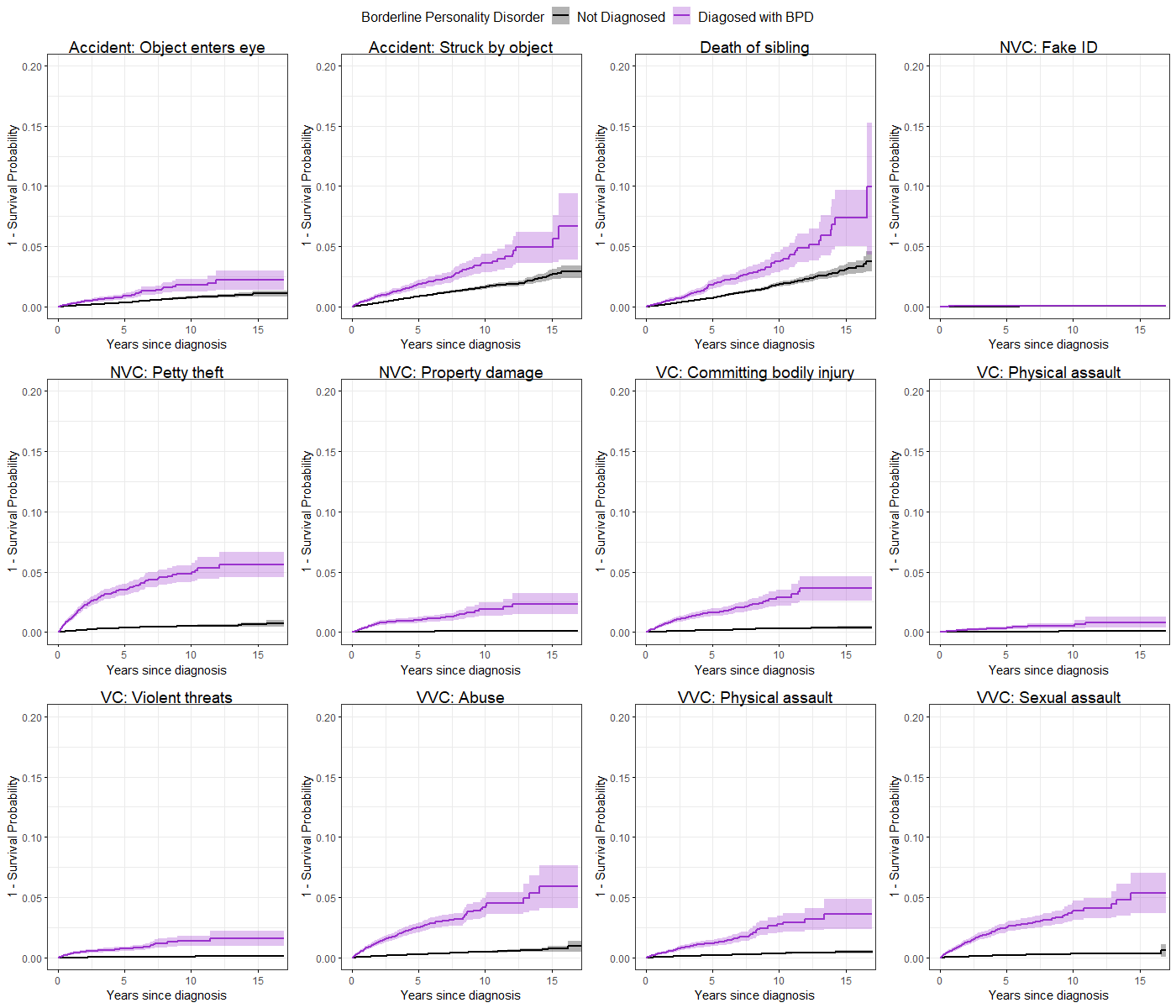


^1^ Abbreviations: Victim of a violent crime requiring medical attention (VVC), Violent crime conviction (VC), Nonviolent crime conviction (NVC)

**Supplementary Figure 24. Associations with Borderline Personality Disorder Diagnosis separated by sex, hazard ratio (95% confidence interval)**
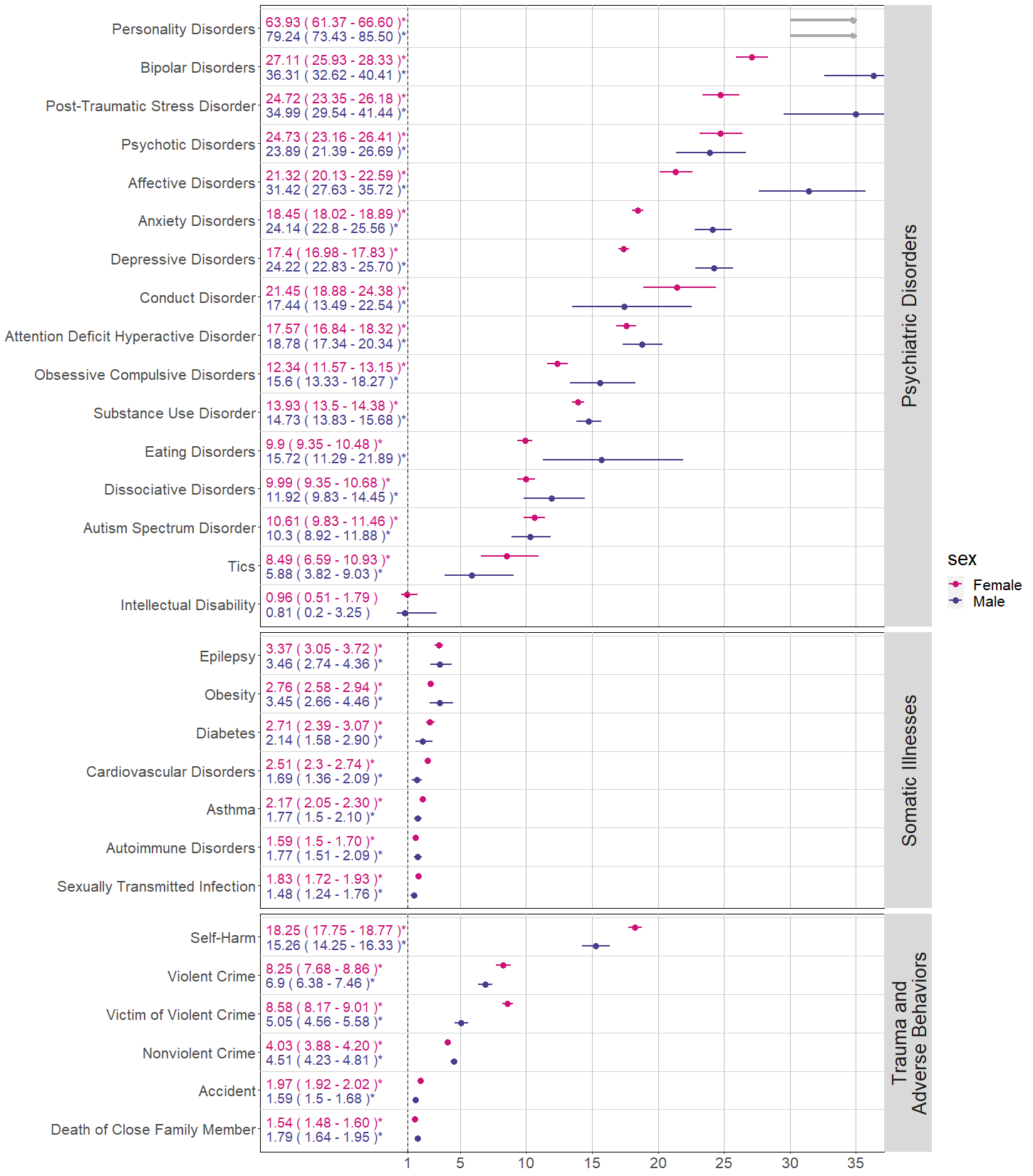


*Statistically significant after correcting for multiple testing

**Supplementary Table 9. Associations with having a sibling with Borderline Personality Disorder Diagnosis and a psychiatric disorder separated by sex, hazard ratio (95% confidence interval)**

|  | **Brother diagnosed   with BPD**  **Female** | | **Brother diagnosed   with BPD**  **Male** | | **Sister diagnosed   with BPD**  **Female** | | **Sister diagnosed   with BPD**  **Male** | |
| --- | --- | --- | --- | --- | --- | --- | --- | --- |
| Affective Disorders | 3.35 | ( 2.18 - 5.14 )* | 1.94 | ( 0.93 - 4.08 ) | 3.27 | ( 2.73 - 3.92 )* | 3.38 | ( 2.69 - 4.26 )* |
| Anxiety Disorders | 3.08 | ( 2.65 - 3.58 )* | 4.00 | ( 3.36 - 4.75 )* | 2.69 | ( 2.52 - 2.88 )* | 2.47 | ( 2.26 - 2.70 )* |
| Attention-Deficit Hyperactive Disorder | 4.66 | ( 3.58 - 6.08 )* | 3.69 | ( 2.86 - 4.76 )* | 3.25 | ( 2.86 - 3.70 )* | 2.74 | ( 2.44 - 3.07 )* |
| Autism Spectrum Disorder | 2.87 | ( 1.73 - 4.77 )* | 2.86 | ( 1.91 - 4.27 )* | 3.31 | ( 2.73 - 4.02 )* | 2.24 | ( 1.88 - 2.68 )* |
| Bipolar Disorders | 6.22 | ( 4.79 - 8.07 )* | 3.80 | ( 2.39 - 6.04 )* | 3.66 | ( 3.18 - 4.22 )* | 3.52 | ( 2.89 - 4.30 )* |
| Conduct Disorder | 4.03 | ( 1.51 - 10.77 )* | 4.05 | ( 1.81 - 9.02 )* | 5.27 | ( 3.71 - 7.50 )* | 2.81 | ( 1.94 - 4.09 )* |
| Depressive Disorders | 3.01 | ( 2.58 - 3.52 )* | 3.63 | ( 3.00 - 4.38 )* | 2.71 | ( 2.53 - 2.90 )* | 2.80 | ( 2.57 - 3.05 )* |
| Dissociative Disorders | 2.65 | ( 1.73 - 4.06 )* | 4.45 | ( 2.76 - 7.16 )* | 2.17 | ( 1.78 - 2.65 )* | 1.64 | ( 1.20 - 2.25 )* |
| Eating Disorders | 2.40 | ( 1.64 - 3.50 )* | 0.88 | ( 0.12 - 6.28 ) | 1.79 | ( 1.50 - 2.14 )* | 1.17 | ( 0.61 - 2.25 ) |
| Intellectual Disability | 0.00 | ( 0.00 - Inf ) | 0.00 | ( 0.00 - Inf ) | 1.57 | ( 0.75 - 3.31 ) | 0.95 | ( 0.42 - 2.11 ) |
| Obsessive Compulsive Disorders | 2.64 | ( 1.68 - 4.14 )* | 4.82 | ( 3.17 - 7.33 )* | 2.45 | ( 2.02 - 2.97 )* | 2.49 | ( 1.98 - 3.15 )* |
| Personality Disorders (PD) | 4.50 | ( 3.25 - 6.25 )* | 5.78 | ( 4.01 - 8.33 )* | 4.34 | ( 3.77 - 5.00 )* | 3.67 | ( 3.03 - 4.43 )* |
| PD: Antisocial | 10.00 | ( 2.48 - 40.34 )* | 3.01 | ( 0.75 - 12.08 ) | 6.26 | ( 2.94 - 13.36 )* | 3.29 | ( 1.90 - 5.71 )* |
| PD: Avoidant | 3.61 | ( 1.16 - 11.21 )* | 5.24 | ( 1.68 - 16.32 )* | 3.40 | ( 2.07 - 5.59 )* | 1.44 | ( 0.60 - 3.49 ) |
| PD: Not Specified | 4.90 | ( 3.44 - 6.97 )* | 6.75 | ( 4.48 - 10.18 )* | 4.66 | ( 3.99 - 5.43 )* | 3.78 | ( 3.01 - 4.74 )* |
| Post-Traumatic Stress Disorder | 4.09 | ( 2.72 - 6.16 )* | 6.76 | ( 3.74 - 12.24 )* | 3.62 | ( 3.02 - 4.35 )* | 2.62 | ( 1.78 - 3.87 )* |
| Psychotic Disorders | 4.29 | ( 2.73 - 6.73 )* | 3.59 | ( 2.36 - 5.46 )* | 2.46 | ( 1.92 - 3.15 )* | 2.55 | ( 2.08 - 3.12 )* |
| Psychotic: Schizoaffective Disorder | 2.85 | ( 0.71 - 11.44 ) | 1.58 | ( 0.22 - 11.25 ) | 3.02 | ( 1.70 - 5.34 )* | 4.35 | ( 2.65 - 7.16 )* |
| Psychotic: Schizophrenia | 5.61 | ( 2.51 - 12.53 )* | 5.03 | ( 2.70 - 9.37 )* | 2.32 | ( 1.37 - 3.93 )* | 3.25 | ( 2.35 - 4.48 )* |
| Psychotic: Schizotypal^1^ | NA |  | 6.99 | ( 1.74 - 28.12 )* | 3.10 | ( 1.15 - 8.35 )* | 4.16 | ( 1.96 - 8.82 )* |
| Substance Use Disorder | 3.54 | ( 2.91 - 4.30 )* | 3.22 | ( 2.68 - 3.86 )* | 2.92 | ( 2.67 - 3.19 )* | 2.06 | ( 1.88 - 2.25 )* |
| Tics | 1.85 | ( 0.26 - 13.14 ) | 2.49 | ( 0.93 - 6.64 ) | 3.34 | ( 1.84 - 6.08 )* | 1.67 | ( 1.05 - 2.67 )* |

*Statistically significant after correcting for multiple testing

^1^No cases for females with a brother diagnosed with BPD

**Supplementary Table 10. Associations with having a sibling with Borderline Personality Disorder Diagnosis and a somatic illness separated by sex, hazard ratio (95% confidence interval)**

|  | **Brother diagnosed   with BPD**  **Female** | | **Brother diagnosed   with BPD**  **Male** | | **Sister diagnosed   with BPD**  **Female** | | **Sister diagnosed   with BPD**  **Male** | |
| --- | --- | --- | --- | --- | --- | --- | --- | --- |
| Asthma | 1.89 | ( 1.51 - 2.37 )* | 1.27 | ( 0.95 - 1.70 ) | 1.46 | ( 1.31 - 1.62 )* | 1.33 | ( 1.19 - 1.48 )* |
| Autoimmune Disorders | 1.14 | ( 0.88 - 1.48 ) | 1.28 | ( 0.95 - 1.70 ) | 1.18 | ( 1.06 - 1.31 )* | 1.12 | ( 0.99 - 1.26 ) |
| Autoimmune: Intestinal Malabsoprtion | 1.38 | ( 0.76 - 2.50 ) | 1.94 | ( 0.92 - 4.07 ) | 1.21 | ( 0.93 - 1.56 ) | 1.08 | ( 0.73 - 1.58 ) |
| Autoimmune: Psoriasis | 1.04 | ( 0.56 - 1.93 ) | 0.86 | ( 0.41 - 1.80 ) | 1.34 | ( 1.07 - 1.68 )* | 1.17 | ( 0.90 - 1.51 ) |
| Autoimmune: Ulcerative Colitis | 0.99 | ( 0.45 - 2.21 ) | 0.79 | ( 0.33 - 1.90 ) | 1.04 | ( 0.75 - 1.44 ) | 0.93 | ( 0.67 - 1.30 ) |
| Cardiovascular Disorders (CVD) | 1.66 | ( 1.14 - 2.42 )* | 1.21 | ( 0.81 - 1.78 ) | 1.25 | ( 1.04 - 1.50 )* | 1.18 | ( 1.01 - 1.39 ) |
| CVD: cardiac arrhythmias | 0.73 | ( 0.10 - 5.20 ) | 0.00 | ( 0.00 - inf ) | 1.40 | ( 0.78 - 2.54 ) | 0.92 | ( 0.44 - inf ) |
| CVD: Paroxysmal tachycardia | 1.75 | ( 0.66 - 4.67 ) | 2.20 | ( 0.82 - 5.86 ) | 1.20 | ( 0.73 - 1.96 ) | 0.87 | ( 0.47 - 1.63 ) |
| CVD: Rheumatic Heart Diseases | 2.01 | ( 1.01 - 4.03 ) | 1.14 | ( 0.48 - 2.75 ) | 1.35 | ( 0.94 - 1.94 ) | 1.49 | ( 1.08 - 2.05 )* |
| Diabetes | 0.99 | ( 0.47 - 2.07 ) | 1.42 | ( 0.80 - 2.50 ) | 1.50 | ( 1.17 - 1.93 )* | 1.26 | ( 0.99 - 1.60 ) |
| Diabetes: Type 1 Diabetes | 0.98 | ( 0.44 - 2.17 ) | 1.48 | ( 0.82 - 2.67 ) | 1.44 | ( 1.10 - 1.90 )* | 1.03 | ( 0.77 - 1.37 ) |
| Diabetes: Type II Diabetes | 1.39 | ( 0.45 - 4.30 ) | 2.24 | ( 0.93 - 5.39 ) | 2.46 | ( 1.71 - 3.53 )* | 2.22 | ( 1.54 - 3.20 )* |
| Epilepsy | 1.18 | ( 0.65 - 2.13 ) | 1.41 | ( 0.82 - 2.43 ) | 1.57 | ( 1.27 - 1.94 )* | 1.53 | ( 1.24 - 1.88 )* |
| Obesity | 1.52 | ( 1.11 - 2.07 )* | 1.42 | ( 0.76 - 2.64 ) | 1.53 | ( 1.35 - 1.75 )* | 1.96 | ( 1.59 - 2.42 )* |
| Sexually Transmitted Infection (STI) | 1.40 | ( 1.11 - 1.77 )* | 1.17 | ( 0.87 - 1.58 ) | 1.18 | ( 1.06 - 1.30 )* | 1.04 | ( 0.91 - 1.18 ) |
| STI: Chlamydia | 1.30 | ( 0.77 - 2.19 ) | 1.14 | ( 0.67 - 1.92 ) | 1.15 | ( 0.92 - 1.45 ) | 0.89 | ( 0.70 - 1.13 ) |
| STI: Herpes Viral Infection | 1.31 | ( 0.87 - 1.97 ) | 0.73 | ( 0.24 - 2.27 ) | 1.21 | ( 1.02 - 1.44 )* | 1.21 | ( 0.85 - 1.74 ) |
| STI: Veneral Warts | 1.39 | ( 1.00 - 1.93 ) | 1.23 | ( 0.84 - 1.79 ) | 1.10 | ( 0.94 - 1.28 ) | 1.00 | ( 0.85 - 1.19 ) |

*Statistically significant after correcting for multiple testing

**Supplementary Table 11. Associations with having a sibling with Borderline Personality Disorder Diagnosis and trauma and adverse behaviors separated by sex, hazard ratio (95% confidence interval)**

|  | **Brother diagnosed   with BPD**  **Female** | | **Brother diagnosed   with BPD**  **Male** | | **Sister diagnosed   with BPD**  **Female** | | **Sister diagnosed   with BPD**  **Male** | |
| --- | --- | --- | --- | --- | --- | --- | --- | --- |
| Accident | 1.36 | ( 1.22 - 1.52 )* | 1.21 | ( 1.11 - 1.33 )* | 1.20 | ( 1.14 - 1.26 )* | 1.06 | ( 1.02 - 1.11 )* |
| Accident: Fall | 1.29 | ( 1.12 - 1.47 )* | 1.24 | ( 1.10 - 1.39 )* | 1.11 | ( 1.05 - 1.18 )* | 1.03 | ( 0.98 - 1.08 ) |
| Accident: Object Enters Eye | 2.03 | ( 1.18 - 3.50 )* | 1.38 | ( 0.96 - 1.97 ) | 1.33 | ( 1.01 - 1.75 ) | 0.94 | ( 0.79 - 1.12 ) |
| Accident: Struck by Object | 1.51 | ( 1.00 - 2.30 ) | 1.59 | ( 1.24 - 2.03 )* | 1.39 | ( 1.16 - 1.66 )* | 1.16 | ( 1.03 - 1.30 )* |
| Death of Close Family Member | 2.24 | ( 1.97 - 2.54 )* | 2.27 | ( 2.00 - 2.58 )* | 1.65 | ( 1.56 - 1.76 )* | 1.66 | ( 1.56 - 1.76 )* |
| Death of Father | 1.97 | ( 1.65 - 2.36 )* | 1.87 | ( 1.55 - 2.24 )* | 1.55 | ( 1.42 - 1.68 )* | 1.65 | ( 1.52 - 1.78 )* |
| Death of Mother | 2.19 | ( 1.71 - 2.79 )* | 1.83 | ( 1.40 - 2.38 )* | 1.42 | ( 1.25 - 1.61 )* | 1.36 | ( 1.20 - 1.55 )* |
| Death of Sibling | 3.60 | ( 2.98 - 4.36 )* | 4.46 | ( 3.75 - 5.29 )* | 2.19 | ( 1.98 - 2.43 )* | 2.09 | ( 1.89 - 2.32 )* |
| Nonviolent Crime | 2.61 | ( 2.20 - 3.10 )* | 2.56 | ( 2.26 - 2.91 )* | 1.93 | ( 1.78 - 2.09 )* | 1.86 | ( 1.75 - 1.97 )* |
| NVC: Fake Passports or Identification | 1.14 | ( 0.54 - 2.38 ) | 0.51 | ( 0.13 - 2.06 ) | 1.30 | ( 0.98 - 1.72 ) | 0.86 | ( 0.56 - 1.31 ) |
| NVC: Petty Theft | 2.52 | ( 2.02 - 3.14 )* | 2.61 | ( 2.06 - 3.30 )* | 1.82 | ( 1.63 - 2.02 )* | 1.86 | ( 1.66 - 2.09 )* |
| NVC: Property Damage | 5.90 | ( 3.49 - 9.98 )* | 3.24 | ( 2.62 - 4.00 )* | 3.26 | ( 2.42 - 4.38 )* | 2.18 | ( 1.96 - 2.42 )* |
| Self-Harm | 3.46 | ( 2.87 - 4.17 )* | 2.81 | ( 2.27 - 3.49 )* | 2.81 | ( 2.58 - 3.06 )* | 2.04 | ( 1.85 - 2.26 )* |
| VC: Assault | 3.37 | ( 1.26 - 9.00 )* | 3.04 | ( 1.91 - 4.82 )* | 1.84 | ( 1.06 - 3.18 )* | 2.89 | ( 2.38 - 3.51 )* |
| VC: Committing Bodily Injury | 2.79 | ( 1.71 - 4.56 )* | 3.12 | ( 2.57 - 3.79 )* | 2.91 | ( 2.38 - 3.56 )* | 1.97 | ( 1.79 - 2.17 )* |
| VC: Violent Threats | 2.50 | ( 0.80 - 7.76 ) | 4.69 | ( 3.55 - 6.22 )* | 2.37 | ( 1.47 - 3.83 )* | 2.76 | ( 2.38 - 3.20 )* |
| Victim of Violent Crime | 2.98 | ( 2.23 - 3.96 )* | 2.07 | ( 1.65 - 2.60 )* | 2.95 | ( 2.61 - 3.32 )* | 1.57 | ( 1.42 - 1.74 )* |
| Violent Crime | 2.84 | ( 1.85 - 4.36 )* | 3.10 | ( 2.61 - 3.69 )* | 3.00 | ( 2.52 - 3.57 )* | 2.10 | ( 1.93 - 2.29 )* |
| VVC: Abuse | 4.31 | ( 2.86 - 6.49 )* | 1.96 | ( 1.40 - 2.75 )* | 3.66 | ( 3.04 - 4.40 )* | 1.62 | ( 1.40 - 1.88 )* |
| VVC: Physical Assault | 2.14 | ( 1.07 - 4.28 )* | 2.24 | ( 1.65 - 3.04 )* | 2.33 | ( 1.77 - 3.07 )* | 1.49 | ( 1.28 - 1.73 )* |
| VVC: Sexual Assault^1^ | 2.07 | ( 1.08 - 3.99 )* | NA |  | 3.49 | ( 2.83 - 4.29 )* | 3.08 | ( 0.76 - 12.50 ) |

^1^No cases of sexual assault for males with a brother diagnosed with BPD

**Supplementary Figure 25. Psychiatric disorders as a risk factor and outcome for a Borderline Personality Disorder diagnosis, hazard ratio (95% confidence interval) ^1,2^**


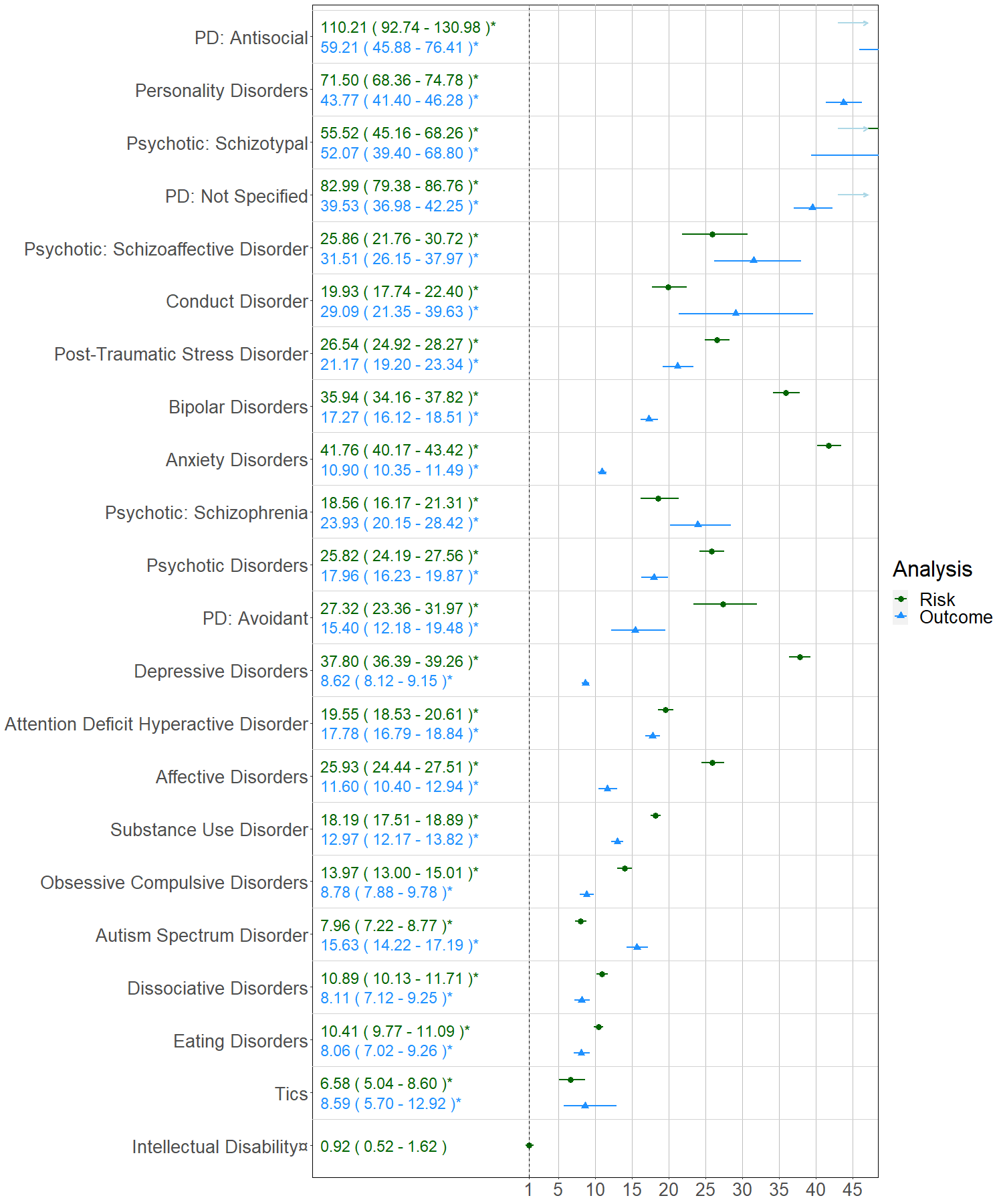


^1^ Risk indicates that the diagnosis occurred before a BPD diagnosis, outcome indicates that the diagnosis occurred after a BPD diagnosis

^2^ Abbreviation: Personality Disorders (PD)

*Statistically significant after correcting for multiple testing

¤Outcome non-estimable

**Supplementary Table 12. Psychiatric disorders as a risk factor or outcome for a Borderline Personality Disorder diagnosis, hazard ratio (95% confidence interval)**

|  | **Males**  **Risk factor prior to BPD diagnosis** | | **Females**  **Risk factor prior to BPD diagnosis** | | **Males**  **Outcomes following BPD diagnosis** | | **Females**  **Outcomes following BPD diagnosis** | |
| --- | --- | --- | --- | --- | --- | --- | --- | --- |
| Affective disorders | 37.42 | (31.77 - 44.08) ^*^ | 24.66 | (23.15 - 26.28) ^*^ | 19.35 | (15.04 - 24.88) ^*^ | 10.70 | (9.48 - 12.08) ^*^ |
| Anxiety | 51.92 | (46.97 - 57.39) ^*^ | 40.10 | (38.45 - 41.82) ^*^ | 13.90 | (12.17 - 15.88) ^*^ | 10.46 | (9.88 - 11.07) ^*^ |
| Attention-deficit hyperactive disorder | 22.55 | (19.93 - 25.52) ^*^ | 19.16 | (18.06 - 20.33) ^*^ | 20.02 | (17.51 - 22.88) ^*^ | 17.14 | (16.08 - 18.27) ^*^ |
| Autism spectrum disorder | 6.69 | (5.29 - 8.46) ^*^ | 8.41 | (7.55 - 9.36) ^*^ | 17.58 | (14.51 - 21.31) ^*^ | 15.03 | (13.47 - 16.76) ^*^ |
| Bipolar disorders | 47.4 | (41.03 - 54.77) ^*^ | 34.51 | (32.68 - 36.44) ^*^ | 25.71 | (21.38 - 30.92) ^*^ | 16.23 | (15.07 - 17.48) ^*^ |
| Conduct disorder | 15.05 | (10.98 - 20.62) ^*^ | 21.13 | (18.64 - 23.96) ^*^ | 36.93 | (21.66 - 62.95) ^*^ | 26.22 | (18.00 - 38.20) ^*^ |
| Depressive disorders | 53.35 | (48.31 - 58.9) ^*^ | 35.57 | (34.14 - 37.06) ^*^ | 10.93 | (9.32 - 12.81) ^*^ | 8.35 | (7.82 - 8.90) ^*^ |
| Dissociative disorders | 13.24 | (10.55 - 16.62) ^*^ | 10.65 | (9.87 - 11.49) ^*^ | 8.93 | (5.93 - 13.47) ^*^ | 7.99 | (6.96 - 9.17) ^*^ |
| Eating Disorders | 14.67 | (10.14 - 21.22) ^*^ | 10.24 | (9.60 - 10.92) ^*^ | 42.36 | (19.90 - 90.17) ^*^ | 7.94 | (6.90 - 9.14) ^*^ |
| Intellectual disability^1^ | 0.83 | (0.21 - 3.32) | 0.95 | (0.51 - 1.76) | NA |  | NA |  |
| Obsessive compulsive disorder | 16.44 | (13.41 - 20.16) ^*^ | 13.63 | (12.62 - 14.71) ^*^ | 11.73 | (8.74 - 15.74) ^*^ | 8.41 | (7.49 - 9.44) ^*^ |
| Personality disorders (PD) | 85.65 | (76.02 - 96.50) ^*^ | 69.33 | (66.05 - 72.77) ^*^ | 67.33 | (59.52 - 76.16) ^*^ | 39.78 | (37.38 - 42.33) ^*^ |
| PD: Antisocial ^2^ | 129.41 | (99.83 - 167.75) ^*^ | 101.92 | (80.78 - 128.58) ^*^ | 47.39 | (31.45 - 71.41) ^*^ | 72.00 | (51.16 - 101.34) ^*^ |
| PD: Avoidant | 33.24 | (22.24 – 22.20) | 26.40 | (22.30 - 31.30) | 29.70 | (18.10 - 48.90) ^*^ | 13.30 | (10.20 - 17.30) ^*^ |
| PD: not specified ^3^ | 113.00 | (100.30 - 127.20) | 79.00 | (75.30 - 82.90) | NA |  | 35.50 | (33.00 - 38.20) ^*^ |
| Post-traumatic stress disorder | 38.93 | (31.78 - 47.69) ^*^ | 25.57 | (23.93 - 27.32) ^*^ | 29.08 | (20.78 - 40.70) * | 20.19 | (18.23 - 22.35) ^*^ |
| Psychotic disorders | 25.4 | (21.92 - 29.42) ^*^ | 25.99 | (24.16 - 27.95) ^*^ | 23.19 | (19.15 - 28.08) ^*^ | 16.57 | (14.71 - 18.66) ^*^ |
| Psychotic: Schizophrenia | 14.01 | (10.4 - 18.86) ^*^ | 20.40 | (17.46 - 23.83) ^*^ | 37.16 | (19.74 - 69.97) ^*^ | 22.99 | (18.58 - 28.44) ^*^ |
| Psychotic: Schizotypal disorder | 38.27 | (23.00 - 63.67) ^*^ | 60.90 | (48.58 - 76.33) ^*^ | 26.88 | (20.06 - 36.03) ^*^ | 56.50 | (41.07 - 77.73) ^*^ |
| Psychotic: Schizoaffective disorder | 36.85 | (25.65 - 52.94) ^*^ | 23.75 | (19.52 - 28.90) ^*^ | 33.27 | (21.73 - 50.94) ^*^ | 31.06 | (25.23 - 38.25) ^*^ |
| Substance use disorders | 22.85 | (20.77 - 25.14) ^*^ | 17.42 | (16.71 - 18.16) ^*^ | 11.91 | (10.24 - 13.84) ^*^ | 12.98 | (12.10 - 13.92) ^*^ |
| Tic disorder | 3.45 | (1.79 - 6.64) ^*^ | 8.35 | (6.23 - 11.20) ^*^ | 8.30 | (3.71 - 18.54) ^*^ | 8.75 | (5.43 - 14.08) ^*^ |

*Indicates significance after correcting for multiple testing

^1^No cases of an intellectual disability diagnosis following a BPD diagnosis

^2^Antisocial personality disorder analysis was modified to exclude males born between 1978 -1982 and women born between 1988.1994 due to high instances of individuals receiving comorbid diagnosis. This led to uninterpretable and non-converging results

^3.^A majority of males were diagnosed with personality disorder not specified which led to an uninterpretable and non-converging result

**Supplementary Figure 26. Somatic illnesses as a risk factor and outcome for a Borderline Personality Disorder diagnosis, hazard ratio (95% confidence interval) ^1,2^**


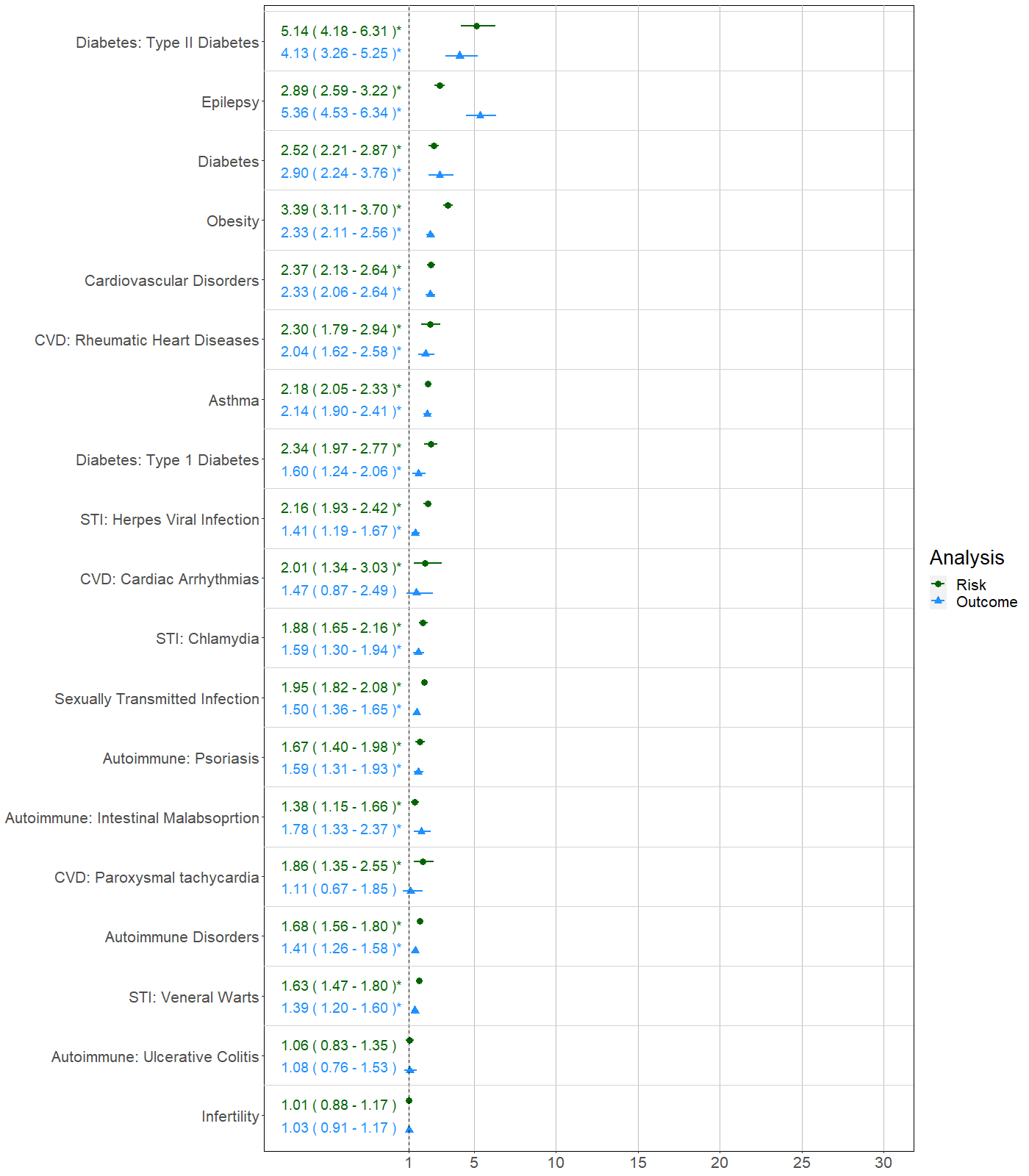


^1^ Risk indicates that the diagnosis occurred before a BPD diagnosis, outcome indicates that the diagnosis occurred after a BPD diagnosis

*Statistically significant after correcting for multiple testing

^2^ Abbreviations: Cardiovascular disorders (CVD),

Sexually transmitted infection (STI)

**Supplementary Table 13. Somatic illnesses as a risk factor or outcome for a Borderline Personality Disorder, hazard ratio (95% confidence interval)**

|  | **Males**  **Risk factor prior to BPD diagnosis** | | **Females**  **Risk factor prior to BPD diagnosis** | | **Males**  **Outcomes following BPD diagnosis** | | **Females**  **Outcomes following BPD diagnosis** | |
| --- | --- | --- | --- | --- | --- | --- | --- | --- |
| Asthma | 1.82 | (1.49 - 2.22) ^*^ | 2.24 | (2.09 - 2.40) ^*)^ | 2.23 | (1.53 - 3.25) ^*^ | 2.18 | (1.92 - 2.47) ^*^ |
| Autoimmune disorders | 1.86 | (1.53 - 2.27) ^*^ | 1.65 | (1.53 - 1.78) ^*^ | 1.61 | (1.16 - 2.22) ^*^ | 1.38 | (1.22 - 1.56) ^*^ |
| Autoimmune: Intestinal malabsorption | 2.26 | (1.28 - 3.99) ^*^ | 1.31 | (1.08 - 1.59) ^*^ | 1.93 | (0.62 - 5.98) | 1.78 | (1.32 - 2.4) ^*^ |
| Autoimmune: Ulcerative Colitis | 1.28 | (0.73 - 2.26) | 1.72 | (1.43 - 2.06) ^*^ | 1.49 | (0.88 - 2.51) | 1.62 | (1.31 – 2.00) ^*^ |
| Autoimmune: Psoriasis | 1.57 | (0.96 - 2.58) | 0.96 | (0.72 - 1.27) | 1.38 | (0.62 - 3.08) | 1.01 | (0.68 - 1.50) |
| Diabetes | 2.15 | (1.52 - 3.03) ^*^ | 2.59 | (2.25 - 2.99) ^*^ | 2.29 | (1.19 - 4.40) ^*^ | 3.05 | (2.30 - 4.04) ^*^ |
| Diabetes: Type II | 4.96 | (2.92 - 8.4) ^*^ | 5.17 | (4.14 - 6.47) ^*^ | 3.3 | (1.78 - 6.15) ^*^ | 4.34 | (3.35 - 5.62) ^*^ |
| Diabetes: Type I diabetes | 1.92 | (1.21 - 3.06) ^*^ | 2.43 | (2.02 - 2.91) ^*^ | 1.29 | (0.67 - 2.47) ^*^ | 1.68 | (1.28 - 2.21) ^*^ |
| Cardiovascular disorders (CVD) | 1.39 | (1.02 - 1.90) ^*^ | 2.62 | (2.33 - 2.94) ^*^ | 2.18 | (1.62 - 2.94) ^*^ | 2.35 | (2.04 - 2.69) ^*^ |
| CVD: cardiac arrhythmias | 1.27 | (0.32 - 5.07) | 2.14 | (1.39 - 3.28) ^*^ | N* |  | 1.71 | (1.01 - 2.90) |
| CVD: Paroxysmal tachycardia | 0.40 | (0.06 - 2.85) | 2.05 | (1.49 - 2.84) ^*^ | N* |  | 1.26 | (0.76 - 2.10) |
| CVD: Rheumatic heart diseases | 1.93 | (1.00 - 3.73) | 2.37 | (1.82 - 3.11) ^*^ | 1.65 | (0.92 - 2.99) | 2.18 | (1.69 - 2.81) ^*^ |
| Epilepsy | 2.95 | (2.21 - 3.93) ^*^ | 2.88 | (2.56 - 3.24) ^*^ | 6.22 | (4.16 - 9.29) ^*^ | 5.25 | (4.36 - 6.31) ^*^ |
| Infertility | N* |  | 1.01 | (0.87 - 1.16) | N* |  | 1.03 | (0.91 - 1.17) |
| Obesity | 3.45 | (2.44 - 4.88) ^*^ | 3.37 | (3.08 - 3.68) ^*^ | 3.91 | (2.64 - 5.80) ^*^ | 2.27 | (2.06 - 2.51) ^*^ |
| Sexually transmitted infection (STI) | 1.65 | (1.32 - 2.07) ^*^ | 1.97 | (1.84 - 2.12) ^*^ | 1.26 | (0.94 - 1.69) | 1.52 | (1.37 - 1.68) ^*^ |
| STI: Chlamydia | 1.43 | (0.96 - 2.13) | 1.96 | (1.70 - 2.26) ^*^ | 1.26 | (0.74 - 2.12) | 1.60 | (1.28 - 1.99) ^*^ |
| STI: Herpes viral infection | 1.40 | (0.67 - 2.95) | 2.17 | (1.93 - 2.43) ^*^ | 1.71 | (0.85 - 3.41) | 1.38 | (1.16 - 1.65) ^*^ |
| STI: Venereal warts | 1.74 | (1.31 - 2.31) ^*^ | 1.61 | (1.44 - 1.79) ^*^ | 1.08 | (0.72 - 1.63) | 1.46 | (1.25 - 1.70) ^*^ |

^a^ Indicates significance after multiple testing

References

1. Mataix-Cols D, Frans E, Pérez-Vigil A, Kuja-Halkola R, Gromark C, Isomura K, et al. A total-population multigenerational family clustering study of autoimmune diseases in obsessive–compulsive disorder and Tourette’s/chronic tic disorders. Molecular psychiatry. 2018;23(7):1652-8.

2. Sariaslan A, Larsson H, D’Onofrio B, Långström N, Fazel S, Lichtenstein P. Does population density and neighborhood deprivation predict schizophrenia? A nationwide Swedish family-based study of 2.4 million individuals. Schizophrenia bulletin. 2015;41(2):494-502.
